# Overactivity or blockade of transforming growth factor‐β each generate a specific ureter malformation

**DOI:** 10.1002/path.5335

**Published:** 2019-10-01

**Authors:** Filipa M Lopes, Neil A Roberts, Leo AH Zeef, Natalie J Gardiner, Adrian S Woolf

**Affiliations:** ^1^ Division of Cell Matrix Biology and Regenerative Medicine, School of Biological Sciences, Faculty of Biology Medicine and Health University of Manchester Manchester UK; ^2^ The Bioinformatics Core Facility University of Manchester Manchester UK; ^3^ Division of Diabetes, Endocrinology and Gastroenterology, School of Medical Sciences, Faculty of Biology, Medicine and Health University of Manchester Manchester UK; ^4^ Royal Manchester Children's Hospital Manchester University NHS Foundation Trust, Manchester Academic Health Science Centre Manchester UK

**Keywords:** embryo, growth factor, human, malformation, mouse, urothelium

## Abstract

Transforming growth factor‐β (TGFβ) has been reported to be dysregulated in malformed ureters. There exists, however, little information on whether altered TGFβ levels actually perturb ureter development. We therefore hypothesised that TGFβ has functional effects on ureter morphogenesis. *Tgfb1*, *Tgfb2* and *Tgfb3* transcripts coding for TGFβ ligands, as well as *Tgfbr1* and *Tgfbr2* coding for TGFβ receptors, were detected by quantitative polymerase chain reaction in embryonic mouse ureters collected over a wide range of stages. As assessed by *in situ* hybridisation and immunohistochemistry, the two receptors were detected in embryonic urothelia. Next, TGFβ1 was added to serum‐free cultures of embryonic day 15 mouse ureters. These organs contain immature smooth muscle and urothelial layers and their *in vivo* potential to grow and acquire peristaltic function can be replicated in serum‐free organ culture. Such organs therefore constitute a suitable developmental stage with which to define roles of factors that affect ureter growth and functional differentiation. Exogenous TGFβ1 inhibited growth of the ureter tube and generated cocoon‐like dysmorphogenesis. RNA sequencing suggested that altered levels of transcripts encoding certain fibroblast growth factors (FGFs) followed exposure to TGFβ. In serum‐free organ culture exogenous FGF10 but not FGF18 abrogated certain dysmorphic effects mediated by exogenous TGFβ1. To assess whether an endogenous TGFβ axis functions in developing ureters, embryonic day 15 explants were exposed to TGFβ receptor chemical blockade; growth of the ureter was enhanced, and aberrant bud‐like structures arose from the urothelial tube. The muscle layer was attenuated around these buds, and peristalsis was compromised. To determine whether TGFβ effects were limited to one stage, explants of mouse embryonic day 13 ureters, more primitive organs, were exposed to exogenous TGFβ1, again generating cocoon‐like structures, and to TGFβ receptor blockade, again generating ectopic buds. As for the mouse studies, immunostaining of normal embryonic human ureters detected TGFβRI and TGFβRII in urothelia. Collectively, these observations reveal unsuspected regulatory roles for endogenous TGFβ in embryonic ureters, fine‐tuning morphogenesis and functional differentiation. Our results also support the hypothesis that the TGFβ up‐regulation reported in ureter malformations impacts on pathobiology. Further experiments are needed to unravel the intracellular signalling mechanisms involved in these dysmorphic responses. © 2019 The Authors. *The Journal of Pathology* published by John Wiley & Sons Ltd on behalf of Pathological Society of Great Britain and Ireland.

## Introduction

The mammalian ureter connects the kidney with the bladder. Cadherin‐1 (CDH1) is located at urothelial intercellular junctions and in the mature organ uroplakin (UPK) proteins coat the lumenal surface of the pseudostratified urothelium, conferring waterproofing properties 1. The urothelium is surrounded by smooth muscle (SM) cells expressing contractile proteins including α‐SM actin (αSMA) and the intermediate filament desmin 2, 3. The SM is surrounded by adventitial fibrocytes. Between the urothelium and SM lie the lamina propria interstitial cells. The ureter propels urine in a proximal (i.e. near the kidney) to distal direction 3. Contractions are initiated by pacemaker cells near the renal pelvis 4, and peristaltic waves are propagated by Cajal‐like cells 5.

The ureter epithelium originates when the ureteric bud branches from the mesonephric duct 6. The bud elongates and its stalk differentiates into urothelia. Mesenchymal cells condense around the urothelial stalk, differentiating into SM 7. The distal end of the stalk joins the bladder 8. In mice, the bud initiates at embryonic day 10 (E10). The bud elongates and becomes surrounded by condensed mesenchyme. At E13 the latter compartment has differentiated so that the inner cells begin to express SM molecules and the outer cells form adventitia 2. At E15 the primitive urothelium has differentiated into basal and superficial cell layers 2. Over the next few prenatal days the ureter begins to transmit urine generated by the metanephros 7, with the urothelium having three cell layers by E18 2. In humans, the bud initiates at 5 weeks gestation and the 10‐week ureter contains a multi‐layered urothelium surrounded by SM 9.

Human ureter malformations can be visualised upon foetal ultrasonographic screening 10. Mild dilation is detected in 5% of foetuses 11. Most are transient anomalies whereas in other individuals dysmorphic ureters persist postnatally 11. Some of these are secondary to primary diseases that prevent urine flow, such as bladder outflow obstruction 12. Other malformations represent intrinsic defects of ureter morphogenesis, ranging from an absent organ to a ureter which has either an occluded lumen or a patent lumen but with dysfunctional peristalsis. Ureter malformations can co‐exist with dysplastic kidneys containing poorly differentiated and metaplastic cells 12.

Molecules in the transforming growth factor‐β (TGFβ) axis have been detected in both kidney and ureter malformations. In human dysplastic kidneys TGFβ1 immunostaining is prominent in metaplastic SM enveloping dysplastic tubules that themselves express TGFβRI and TGFβRII 13, cell surface receptors activated by TGFβ1‐3 ligands 14. Exposing cultured human dysplastic kidney epithelia to TGFβ1 leads to up‐regulated fibronectin 13. In organ culture of mouse metanephric kidneys, exogenous TGFβ1 inhibits tubule formation, whereas blocking endogenous TGFβ1 enhances tubulogenesis 15. Experimental urinary flow obstruction in foetal sheep generates dysmorphic kidney tubules, with increased TGFβ1, TGFβRI and TGFβRII 16. TGFβ1 has been detected in congenital stenoic ureters and megaureters 17, 18, 19 and ureteric ligation in postnatal rats up‐regulated TGFβ1 and its receptors in the ureter capsule 20.

Given that TGFβ pathway molecules have been reported to be up‐regulated in both human kidney and ureter malformations, we here first examined normal human embryos, immunodetecting both TGFβRI and TGFβRII in developing ureters. Key components of the TGFβ axis were detected in embryonic mouse ureters using RT‐qPCR, *in situ* hybridisation and immunohistochemistry. Hypothesising that TGFβ mediates ureter morphogenesis, we added TGFβ1 to serum‐free organ cultures of mouse E15 ureters. These organs contain immature SM and urothelial layers and their *in vivo* potential to grow and acquire peristaltic function can be replicated in serum‐free organ culture, as demonstrated previously 3 and in this study. E15 ureters thus constitute a suitable stage of development with which to define roles of factors that may perturb or enhance ureter growth and functional differentiation. Exogenous TGFβ1 inhibited growth of the ureter tube and generated cocoon‐like dysmorphogenesis. RNA‐sequencing suggested TGFβ altered levels of numerous transcripts, including *Fgf18* and *Fgf10* that code for fibroblast growth factors (FGFs). Given that little is known about the roles of these molecules in ureter development, we added them to serum‐free embryonic ureter cultures. FGF10 but not FGF18 abrogated certain dysmorphic effects mediated by exogenous TGFβ1. To assess whether an endogenous TGFβ axis operates in developing ureters, E15 explants were exposed to TGFβ receptor blockers. Here, aberrant bud‐like structures arose from the urothelial tube and the rate of peristalsis was decreased.

## Materials and methods

### Ethics

Human tissues, collected after maternal consent and ethical approval (REC 08/H0906/21+5), were provided by the MRC and Wellcome Trust Human Developmental Biology Resource (http://www.hdbr.org/). CD1 wild‐type strain mouse experiments were approved by the University of Manchester ethics committee and UK Home Office (licence PAFFCI44F).

### Organ culture

Embryonic ureters were explanted onto platforms (0.4 μm; Millipore, Watford, UK) and cultured for 6 days 3, 21. Explants were fed DMEM/F12 (D8437, Sigma‐Aldrich, Gillingham, UK) containing insulin–transferrin–selenium (41400045, Gibco, Life Technologies, Paisley, UK) and penicillin–streptomycin (Thermo Fisher Scientific, Paisley, UK). Media were renewed at day 3. In some experiments the following were added: recombinant human TGFβ1 (240‐B; R&D Systems, Minneapolis, MN, USA); TGFβR inhibitor LY2109761 (A8464, Generon, Slough, UK); TGFβR inhibitor SB431542 (04‐0010; Generon); recombinant mouse FGF18 (CYT‐064; ProSpec, Ness‐Ziona, Israel); recombinant mouse FGF10 (6224‐FG‐025, R&D Systems); and 5‐bromo‐2′‐deoxyuridine (BrdU; B5002‐100MG, Sigma‐Aldrich), applied 2 h before harvest. Photographs were taken using inverted light microscope (Leica M80; Leica Microsystems, Milton Keynes, UK). Growth was assessed using ImageJ software (National Institutes of Health, Bethesda, MD, USA). For linear growth, a line drawn down the middle of the ureter tube was measured. To measure ureter tube area, a line was drawn around the epithelium plus SM layer. These two parameters give a more sophisticated view of growth than that based on just one dimension. On day 6, the number of waves of peristalsis initiating in the proximal ureter during 2 min was counted by direct inspection using an Eclipse Ti inverted microscope (Nikon UK, Kingston upon Thames, UK) maintaining explants at 37 °C in 5% CO_2_ 21. Statistical comparisons were made using Student's *t*‐test, Mann–Whitney or Kruskal–Wallis tests, as appropriate, adjusting for multiple comparisons when appropriate.

### RNA sequencing

RNA‐sequencing was undertaken, as described previously 22, 23, 24, 25 and detailed in supplementary material, Supplementary materials and methods. Data was deposited in the ArrayExpress repository (E‐MTAB‐7395).

### Histology, RT‐qPCR and *in situ* hybridisation

Please see supplementary material, Supplementary materials and methods for details.

## Results

### Human ureters

**Figure 1:**
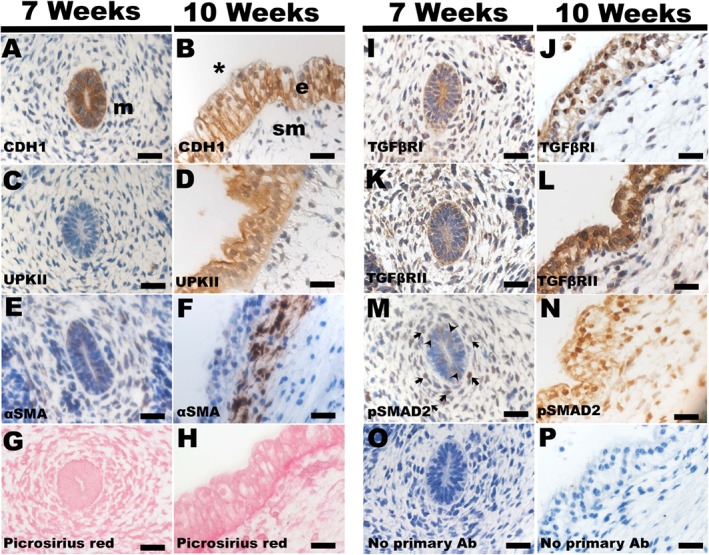
Histology of human embryonic ureters. (A,C,E,G,I,K,M,O) Transverse section of ureter from a 7‐week embryo. (B,D,F,H,J,L,N,P) Longitudinal section of proximal ureter from a 10‐week embryo. All sections were counterstained with haematoxylin (blue) apart from G and H. At 7 weeks CDH1 was detected (brown) in the primitive urothelium (A; *m* indicates mesenchyme), with expression maintained at 10 weeks in the multi‐layered urothelium (B; *sm* indicates SM layer and the asterisk is in the lumen). At 7 weeks UPKII was not immunodetected (C) and αSMA was faintly detected in a subset of mesenchyme‐like cells (D). Both UPKII and αSMA were prominent at 10 weeks (D,F). Pricrosirius red staining showed a faint line around the base of the urothelium at 7 weeks, with a stronger signal in this location at 10 weeks (G,H). TGFβRI and TGFβRII were immunodetected in urothelium at seven (I, K) and 10 weeks (J,L). pSMAD2 was detected in subsets of urothelial cells (*arrowhead* in M) and in subsets of surrounding cells (*arrow* in M) at 7 weeks. pSMAD2 was detected in subsets of urothelial and surrounding cells at 10 weeks (N). No primary antibody negative controls (O,P). Bars, 10 μm.

Ureters in a 7‐week gestation embryo contained epithelial tubes, with walls one to two cells thick, that were immunopositive for CDH1 but not for UPKII, surrounded by mesenchyme‐like cells with αSMA immunostaining barely detectable (Figure 1). Ureters of a 10‐week gestation embryo had urothelium, with multiple layers, immunopositive for CDH1. UPKII immunostaining was detected on the luminal aspect of the urothelial layer, and cells around the urothelium immunostained for αSMA. Picrosirius red, a collagen‐reactive dye 26, stained a basement membrane‐like line around urothelia at 7 weeks, a signal more prominent at 10 weeks. At both ages, epithelia immunostained for TGFβRI and TGFβRII. pSMAD2, a TGFβ canonical intracellular signalling molecule 14 was detected in subsets of epithelia and surrounding cells. Fluorescence immunohistochemistry was undertaken in a separate late first trimester specimen, detecting a plasma membrane‐like localisation of TGFβRI and TGFβRII in urothelia (see supplementary material, Figure S1).

### Mouse ureters *in vivo*

**Figure 2:**
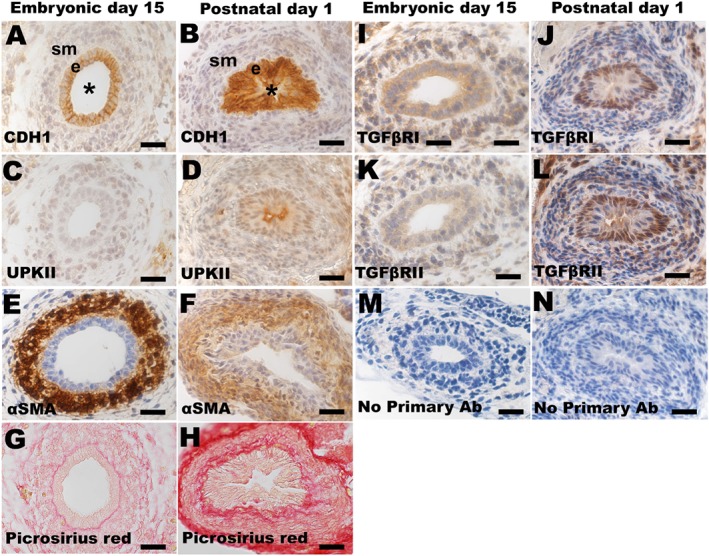
Histology and immunohistochemistry of mouse embryonic ureters. (A,C,E,G,I,K,M) Transverse sections of an E15 ureter within an intact embryo. (B,D,F,H,J,L,N) Transverse sections of ureter from a mouse on the day of birth. All sections were counterstained with haematoxylin (blue) apart from G and H. At E15 CDH1 was detected (brown) in the primitive urothelium (A); *e* indicates the epithelium, *sm* indicates the nascent SM layer, and the asterisk is in the lumen. At this age, UKPII was not detected in the epithelium (C), the surrounding cells expressed αSMA (E), and picrosirius red stained in a linear pattern adjacent to the base of the urothelium (G). In the neonatal ureter, both CDH1 (B) and UPKII (D) were detected in the urothelium, αSMA was detected in the SM layer (F), and picrosirius red staining was prominent in the putative interstitial layer adjacent to the urothelium, and in the adventitia (H). TGFβRI and TGFβRII were immunodetected in the E15 (I,K) and neonatal (J,L) ureter. No primary antibody negative controls (M,N). Bars, 20 μm.

The E15 mouse ureter (Figure 2) consisted of an epithelial tubule, two cells deep, immunostaining for CDH1 but not UPKII. The tube was surrounded by a nascent SM layer expressing αSMA. In the CD1 mice used here, birth occurs at 21 days of gestation i.e. 6 days after E15. Neonates (Figure 2) had a multi‐layered urothelium that was immunopositive for CDH1 and UKPII, surrounded by αSMA expressing SM. Picrosirius red staining revealed a basement membrane‐like patterns on the ablumenal border of the E15 epithelium while neonatally the most prominent signal was in the adventitia. Bright‐field peroxidase‐based immunohistochemistry (Figure 2) showed signals for TGFβRI and TGFβRII in E15 and neonatal ureters. Fluorescence immunohistochemistry at E15 detected a plasma membrane like pattern for TGFβRI and TGFβRII in the urothelium (see supplementary material, Figure S2). We undertook RT‐qPCR for *Tgfb1*, *Tgfb1*, *Tgfb3*, *Tgfbr1* and *Tgfbr2* using mRNA from freshly dissected mouse ureters. These results (see supplementary material, Figure S3), showed that all were expressed over a wide span of mouse ureter development i.e. at embryonic days 13, 15 and 18, and on the day of birth. Given that a focus of our functional experiments were E15 explants, below, *in situ* hybridisation was undertaken for *Tgfb1*, *Tgfbr1* and *Tgfbr2* on E15 ureters (see supplementary material, Figure S4). Sparse signals for *Tgfb1* were detected. More prominent signals were detected for *Tgfbr1* and *Tgfbr2*. Both receptor mRNAs were detected in the epithelium, consistent with the immunohistochemical data for TGFβRI and TGFβRII, showing that this layer is equipped with two key receptors to bind and initiate signalling by TGFβ ligands. *Tgfbr2* was also detected in lamina propria, SM and adventitia.

### TGFβ axis molecules in embryonic ureter culture

As expected 3, 21, E15 explants fed serum‐free control media alone grew over 6 days, elongating an average of 1.2 mm, and increasing in area by an average of 0.3 mm^2^ (see supplementary material, Figure S5). From 2 days, as expected 3, 21, explants displayed spontaneous peristalsis. RNA‐sequencing at one and 6 days confirmed epithelial molecular maturation, with increased levels of transcripts encoding UPKIa, UPKIb, UPKII and UPKIIIa (each *p* < 0.05, corrected for multiple comparisons). Transcripts encoding αSMA were expressed at both times with a non‐significant increase, while transcripts for desmin, a SMC protein up‐regulated later than αSMA *in vivo* in embryonic urinary tracts 27, showed a significant (*p* < 0.05) increase between days 1and 6. We sought transcripts encoding TGFβ axis members in the array (see supplementary material, Table S1). *Tgfb1*, *Tgfb2* and *Tgfb3* were robustly expressed (average reads >100) on days 1 and 6. *Tgfb1* significantly (*p* = 0.003, after adjusting for multiple comparisons) increased during culture, while *Tgfb2* and *Tgfb3* tended to fall. *Tgfbr1* and *Tgfbr2* were robustly expressed, as was *Tgfbr3* encoding TGFβRIII, or betaglycan, a proteoglycan that sequesters TGFβ 28.

### Effects of exogenous TGFβ1

**Figure 3:**
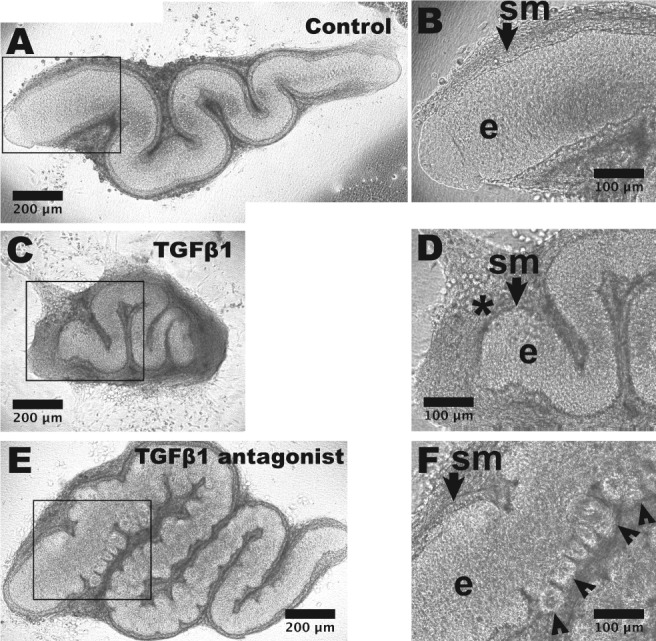
Gross morphology of E15 ureter explants. Explants viewed from above as whole mounts at day 6 of culture. (A) An organ fed basal media only shows a snake‐like appearance, with high power of the proximal part (boxed) shown in (B). The SM layer (*sm*, arrowed) and epithelium (*e*) are indicated. (C) An organ fed with media supplemented with 5 ng/ml TGFβ1. Note that it is smaller than the control organ and it has cocooned appearance. In the high power view (D), the ureter tube is surrounded by a prominent interstitial cell layer (*asterisk*). (E) Organ after 6 days of culture fed with media supplemented with 10 μm LY2109761 that inhibits TGFβRI/TGFβRII kinase. Note the overgrown appearance compared with the control organ, with numerous bud‐like structures protruding from the epithelial tube. Some of these are visualised (arrowheads) in the high power image (F).

**Figure 4:**
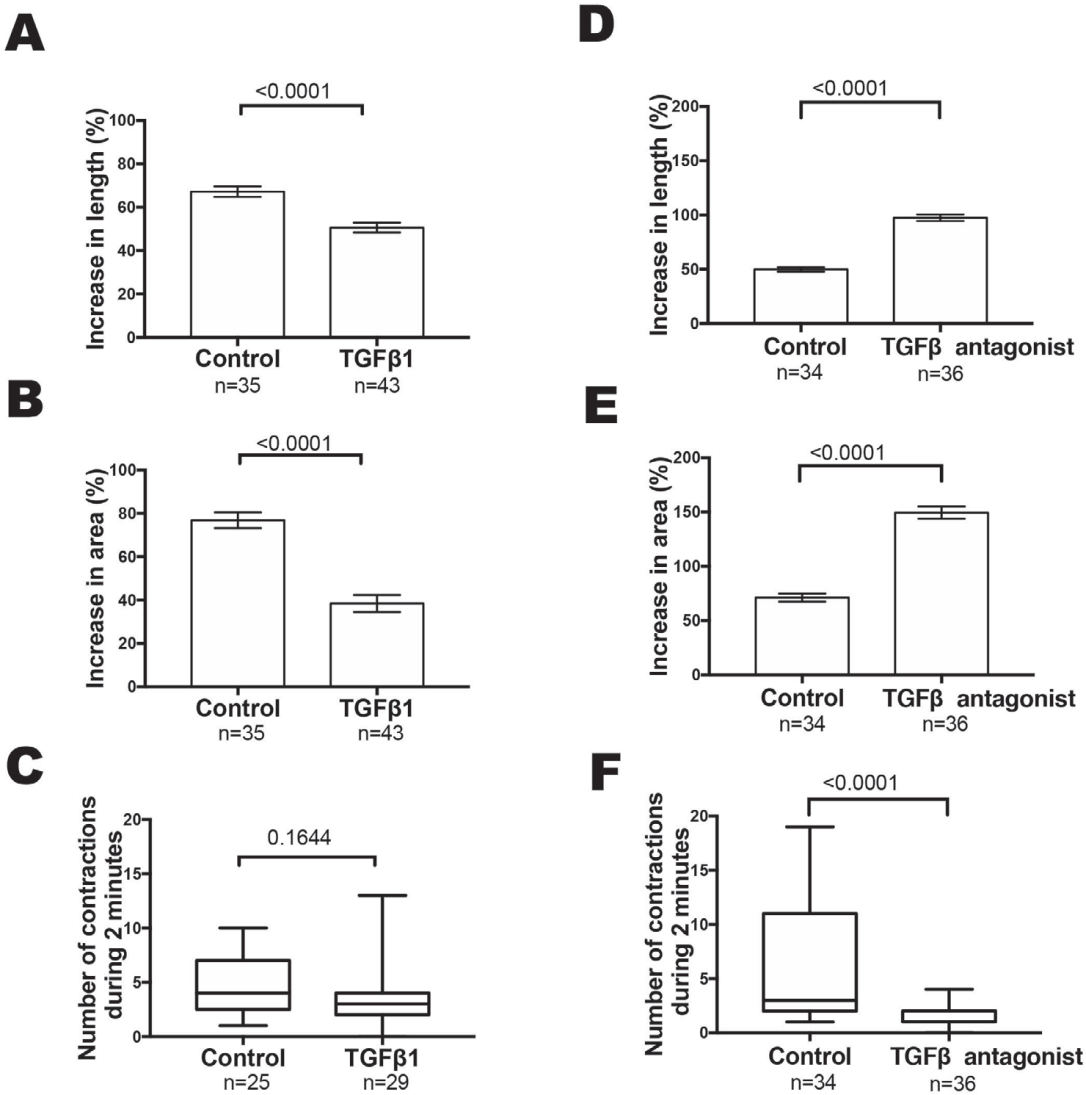
Quantification of growth and peristalsis in E15 ureter explants assessed at day 6 of culture. (A–C) Explants exposed to exogenous 5 ng/ml TGFβ1 showed significantly less (*p* < 0.001) elongation compared with explants fed basal media alone (A). A similar conclusion was made regarding the explant area (*p* < 0.001) (B). In both, growth of each explant was expressed as the percent increase over the length or area of the same explant measured on the day when it was explanted. Exogenous TGFβ1 did not significantly affect the number of contractions measured during 2 min (C). (D,E,F) Organs fed with basal media supplemented with 10 μm LY2109761, a molecule that inhibits TGFβRI/TGFβRII kinase. LY2109761 significantly increased ureter growth assessed as both length (D) and area compared with explants fed basal media alone. LY2109761 significantly decreased the number of ureter contractions recorded during 2 min compared with controls (F). Data are depicted as either mean ± SEM or median, interquartile range and range, as appropriate for the distribution of data points. Numbers of organs assessed are indicated on the graphs under the horizontal axes.

Basal media was supplemented with TGFβ1 at concentrations similar to those used when exploring effects of this molecule on mouse embryonic kidney, small intestine and salivary gland explants 15, 29, 30, 31. About 5 and 50 ng/ml concentrations were each able to alter explant morphology, detailed below. One ng/ml had no overt effect and was not studied further (not shown). After 2 days, 5 ng/ml TGFβ1 exposed explants began to acquire a ‘cocoon’, with a prominent adventitia, a dysmorphic appearance that became more marked by day 6 (Figure 3). Five ng/ml TGFβ1 significantly reduced length and area growth, as assessed on the final day of culture (Figure 4) but these explants underwent peristalsis as normal (Figure 4). Fifty ng/ml TGFβ1 had more marked effects, with day 6 organs resembling circular discs (not shown): these underwent peristalsis, showing they were viable.

To determine whether exogenous TGFβ1 effects were restricted to E15 organs, we also studied E13 rudiments that is less differentiated than E15 organs 2. E13 explants exposed to 5 ng/ml TGFβ1 also acquired a cocoon‐like phenotype (see supplementary material, Figure S6).

**Figure 5:**
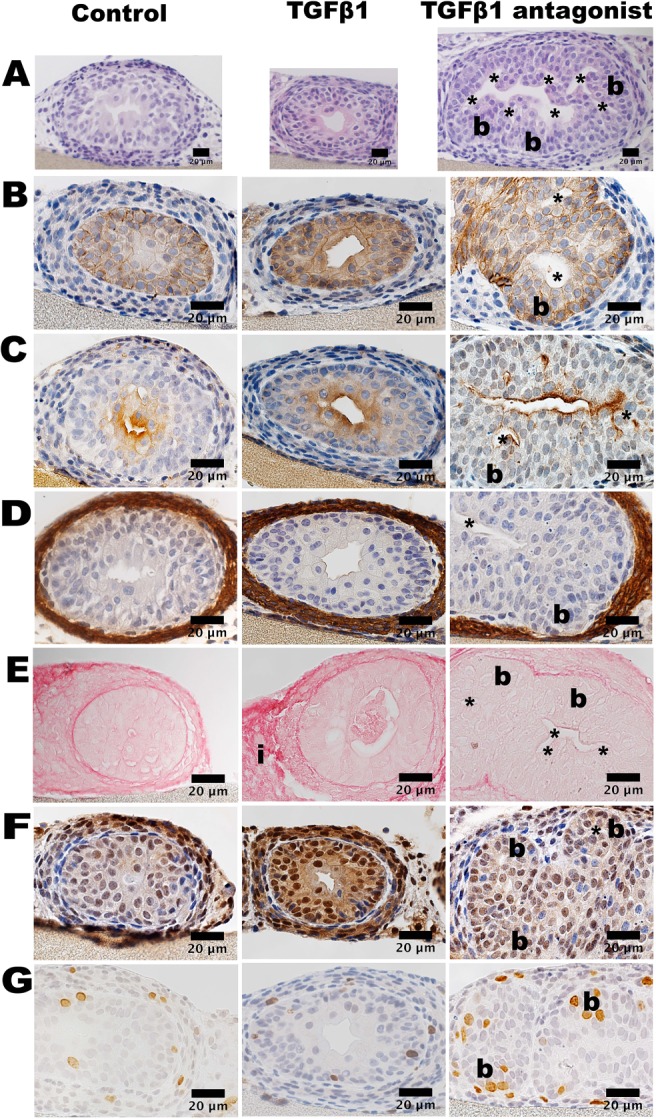
Histology of cultured E15 ureters. The first column depicts sections of organs fed basal media alone (Control); the second depicts transverse sections of organs exposed to exogenous 5 ng/ml TGFβ1 (*TGFβ1*); and the third depicts transverse sections of organs exposed to TGFβ blocker LY2109761 (*TGFβ1 antagonist*). Sections were counterstained with haematoxylin apart from E. (A) Eosin stained sections reveal the smaller profile of the TGFβ1 exposed ureter and the larger profile of the LY2109761 exposed ureter versus control. The lumen in the LY2109761 exposed ureter had extensions (asterisks) from the main lumen. (B) CDH1 and (C) UPKII immunostaining (brown). In the LY2109761 exposed organ note the bud‐like structure (b). (D) In all three conditions, an αSMA immunostained layer (brown) was noted around the urothelium. This layer appeared attenuated over the bud in the LY2109761 exposed ureters. (E) Picrosirius red staining (red) was prominent in adventitial tissue (i) in TGFβ1 exposed organs. (F) pSMAD2 immunostaining (brown) detected positive nuclei in the three conditions. (G) BrdU immunostained (brown) nuclei were detected in both epithelial and surrounding layers in all conditions. In the organ exposed to LY2109761, clusters of positive nuclei were detected in bud‐like structures. Bars, 20 μm.

Hereafter, we mostly focused on E15 rudiments exposed to 5 ng/ml TGFβ1. The histology of dysmorphic explants harvested day 6 (Figure 5) showed prominent adventitia. The SM layer was intact and immunostained for αSMA, as in controls. The urothelium in both control explants and TGFβ1 exposed explants immunostained for CDH1 and UKPII. pSMAD2 immunostaining appeared prominent in TGFβ1 exposed cultures but was not quantified. We undertook proliferation assays with BrdU incorporation after 24 h of culture (see supplementary material, Figure S7), reasoning that any changes found would be less likely to reflect secondary effects from the cocooning that became prominent later on. No significant differences were found between controls and TGFβ1‐exposed organs in either epithelial, SM or adventitial layers.

### FGFs in ureter cultures

We compared RNA‐sequencing datasets in TGFβ1‐exposed and control explants harvested after 24 h. After adjustment for multiple comparisons, levels of several hundred species of transcripts differed significantly. The most significantly changed transcripts are listed in supplementary material, Table S2, with the full set deposited in the ArrayExpress repository (E‐MTAB‐7395). Transcripts considered in the Discussion are annotated in the volcano plot (see supplementary material, Figure S8). Among the up‐regulated transcripts after exposure to 5 ng/ml of TGFβ1, was *Fgf18* (see supplementary material, Table S3). Levels were significantly increased (*p* = 5x10^−12^, average reads 172 versus 27) after 24 h in culture. We undertook RT‐qPCR for *Fgf18*, and confirmed its significant up‐regulation (see supplementary material, Figure S9). RT‐qPCR showed that *Fgf18* was also expressed in native ureters harvested between E13 and birth (see supplementary material, Figure 3). Cultures exposed to exogenous TGFβ1 for 6 days showed a significant increase of *Fgf18* versus time‐matched controls (*p* = 0.03, average reads 143 compared with 8). Reasoning that FGF18 might affect ureter development, E15 explants were studied: with basal media alone; with basal media supplemented with 200 ng/ml FGF18, a concentration effective in chondrocyte proliferation assays 32; with basal media supplemented with TGFβ1; or with both exogenous FGF18 and TGFβ1. Addition of FGF18 alone produced a modest and significant elongation of the ureter tube versus controls. FGF18 did not, however, ameliorate TGFβ1 induced dysmorphogenesis (see supplementary material, Figure S10).

**Figure 6:**
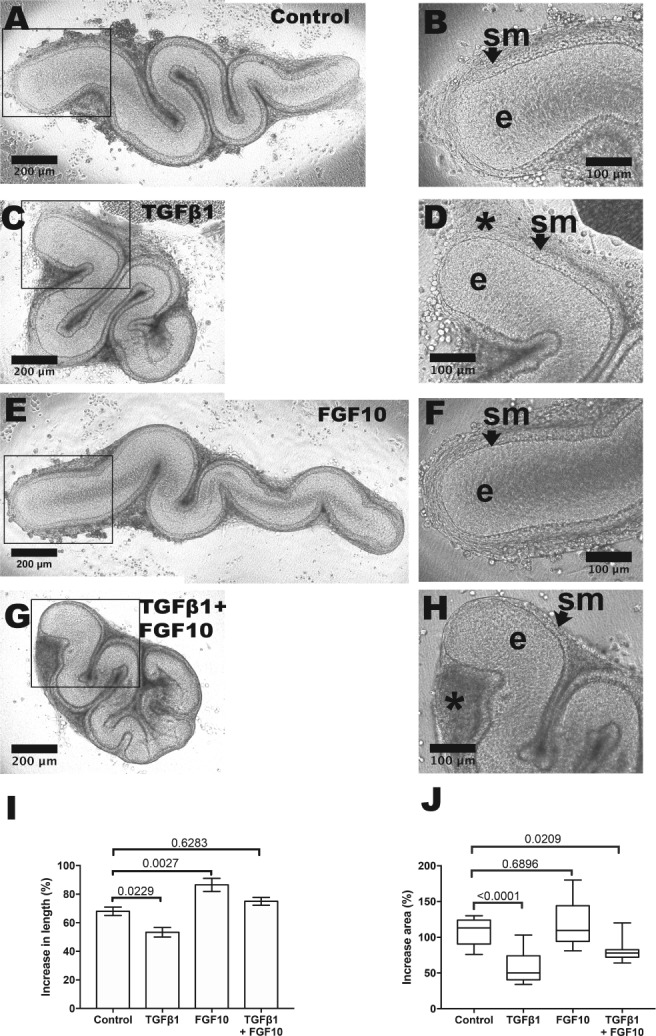
Effects of FGF10 in organ culture. (A–H) Explants viewed at day 6. (A) Organ fed basal media only, with high power of the boxed area shown in (B). SM (*sm*, arrowed) and epithelial (*e*) zones are indicated. (C,D) Organ fed with basal media supplemented with 5 ng/ml TGFβ1 has a cocoon‐like appearance. Interstitial zone indicated by asterisk. (E,F) Organ fed with basal media supplemented with 500 ng/ml FGF10. Note the apparent increased length versus organ fed basal media alone. (G,H) Organ fed with basal media supplemented with both 5 ng/ml TGFβ1 and 500 ng/ml FGF10. Cocooning is still apparent with prominent interstitial tissue (*) but the length of the tube appears increased versus the organ exposed to TGFβ1 alone. (I,J) Quantification of increases in urothelial tube length (I) and area (J) show that FGF10 (*n* = 12) caused significant linear growth versus basal media alone (*n* = 13). Addition of TGFβ1 (*n* = 13) caused significant reduction in linear and area growth versus basal media alone. When FGF10 was added together with TGFβ1 (*n* = 13) the negative effect of the latter on linear growth was overcome.

RNA sequencing of explants after 24 h exposure to TGFβ1 found a numerically modest but statistically significant down‐regulation of *Fgf10*, which encodes FGF10, a recognised urothelial mitogen 33. Although RT‐qPCR showed only a non‐significant tendency for *Fgf10* levels to fall (see supplementary material, Figure S9), we tested whether exogenous FGF10 might affect ureter growth. In these experiments (Figure 6) basal media was supplemented with 500 ng/ml FGF10, a concentration that restores ureteric bud growth in embryonic mouse kidneys with defective receptor tyrosine kinase signalling 34. These cultures showed significantly increased lengths versus controls. In other cultures, media was supplemented with both FGF10 and TGFβ1. Here, although the cocooning effect of TGFβ1 was still evident, the TGFβ1‐induced deceleration in linear growth was overcome. Immunostaining revealed FGF10 (see supplementary material, Figure S11) in human and mouse embryonic ureters *in vivo*, and in explanted mouse E15 ureters. RT‐qPCR showed that *Fgf10* was expressed also in native ureters harvested between E13 and birth (see supplementary material, Figure 3). The 24 h RNA sequence data was interrogated to seek other *Fgf* transcripts 35 and results are shown in supplementary material, Table S3. In controls, *Fgf1*, *Fgf2*, *Fgf7*, *Fgf11*, *Fgf10*, *Fgf13* and *Fgf14* were each robustly expressed (average reads >100), *Fgf5*, *Fgf9*, *Fgf12*, *Fgf18* and *Fgf20* were moderately expressed (average reads 10–100), while *Fgf3*, *Fgf4*, *Fgf6*, *Fgf8*, *Fgf15*, *Fgf16*, *Fgf21*, *Fgf22* and *Fgf23* were barely or not expressed (reads 0–9). Regarding receptors that transduce FGF signals, robust levels (reads >100) of *Fgfr1*, *Fgfr2*, *Fgfr3* and *Fgfr4* were detected in controls.

### TGFβ receptor blockade in ureter culture

As described above, explanted E15 ureters expressed transcripts of *Tgfb1*, *Tgfb2* and *Tgfb3*, as well as from *Tgfbr1* and *Tgfbr2*, the genes coding for their signal transducing receptors. This raised the possibility that endogenous TGFβ ligands affect differentiation. Accordingly, we supplemented basal media with either LY2109761, that inhibits TGFβRI/TGFβRII kinase activity 36, or SB431542, that inhibits TGFβRI kinase 37. LY2109761 (10 μm) resulted in ureter overgrowth, with bud‐like structures initiated from the urothelial tube, most prominent proximally (Figure 3E,F). Inhibition of TGFβ1 also caused enhanced linear and area growth (Figure 4D,E). In E15 ureters exposed to LY2109761, peristalsis was significantly decreased versus controls (Figure 4F). As assessed by histology of day 6 organs (Figure 5), in LY2109761 explants we visualised pocket‐like protrusions from the main lumen terminating in bud‐like structures containing clusters of BrdU positive cells. SM over these buds appeared attenuated (Figure 5D). As assessed by BrdU incorporation, after 24 h of culture (see supplementary material, Figure S7), in E15 organs exposed to TGFβ receptor blockade there was no change in the SM compartment, a significant (*p* = 0.0086) decrease in the adventitial compartment, and an increase approaching significance (*p* = 0.0699) in the epithelial compartment. As for E15 explants, E13 rudiments exposed to LY2109761 also acquired bud‐like structures (see supplementary material, Figure S6), although growth was not formally quantified. SB431542, a different type of TGFβ receptor blocker, also resulted in bud‐like structures in E15 explants, and it caused an increase in ureter tube area when applied at 10 or 20 μm (see supplementary material, Figure S12).

The cocooning effect of exogenous TGFβ1 and its growth decelerating effect on area were partly abrogated (see supplementary material, Figure S13) by LY2109761. This goes some way to prove that the dysmorphic effects of exogenous TGFβ1 were mediated through TGFβRI/TGFβRII.

## Discussion

This study showed that TGFβ axis molecules are detected in both human and mouse developing ureters. Moreover, unique dysmorphic phenotypes were generated by adding exogenous TGFβ1 (i.e. inhibition of growth and generation of a cocoon‐like phenotype) or by adding TGFβ receptor blockers (i.e. acceleration of linear growth accompanied by formation of epithelial ‘buds’).

Previous studies defined molecules directing ureteric bud initiation, branching of its top end, and the connection of its distal end to the bladder. These included secreted molecules such as glial cell line‐derived growth factor (GDNF), bone morphogenetic proteins (BMPs), FGFs and retinoic acid 8, 34, 38, 39. Growth factor control of stalk development has been studied less. The embryonic urothelium secretes sonic hedgehog (SHH) that induces BMP4 in nearby mesenchyme: here BMP4 leads to induction of SM proteins 40, 41. SHH signalling is also required for pacemaker maturation 42, while Cajal‐like cells in the SM layer of the ureteric express KIT, a growth factor receptor needed for their function 5. Before our study, however, little had been reported about the possible roles for TGFβ in the embryonic ureter.

We discovered that blocking endogenous TGFβ, with either of two receptor inhibitors, generated epithelial overgrowth in embryonic ureters, thus revealing a previously unreported regulatory role for the TGFβ axis in morphogenesis of the ureteric stalk. The phenotype may in part be explained by the fact that, based on experiments with cultures of postnatal urothelia, exogenous TGFβ1 inhibits proliferation 43. Thus, in the current context, blockade would be associated with overgrowth, as manifested by aberrant bud‐like structures. The current results observed after blockade of endogenous TGFβ are broadly consistent with the observation that a monoclonal antibody to TGFβ1 administered to pregnant rabbits increased the incidence of ureter malformations in offspring 44. The dysmorphic effects of TGFβ blockade on developing ureters have parallels in other organs. Antibody mediated TGFβ1 blockade causes accelerated nephron tubule formation in metanephric kidney organ culture 15, and inhibiting TGFβRI in embryonic lungs increases branching 45. In contrast to the bud‐like structures observed in the ureter stalk, this lung phenotype appears confined to the distal sections of the bronchial tube, where branching normally occurs 45. The bud‐like phenotype of the ureter upon TGFβR blockade appear morphologically similar to that reported in embryonic ureter stalks exposed to GDNF or FGF7 46. We speculate that these buds, to a small extent, mimic the phenotype of inverted papillomas, a rare human ureter disorder. In this disease, the ureteric urothelium extends cords of epithelial cells in away from the organ's lumen 47, 48. In future, it may be informative to analyse expression of TGFβ axis molecules in these benign tumours.

This study additionally discovered that exogenous TGFβ1 causes embryonic ureter malformations, generating a phenotype distinct from that caused by TGFβ blockade. This supports the idea that an overactive TGFβ axis contributes to dysmorphogenesis in human renal tract malformations 17, 18, 19. Exogenous TGFβ1 caused the explanted ureteric tube to become encased in a cocoon‐like structure. This may have generated a physical constraint limiting ureter tube growth, and indeed we did not find a decreased proliferative index in explants. On the other hand, exogenous TGFβ1 is reported to decrease proliferation of postnatal urothelia 43 and ureteric bud tips 49, and it compromises branching morphogenesis in salivary glands 31, 50 and lung 45. Another study reported that embryonic rat ureters exposed to TGFβ had impaired urothelial and SM proliferation 51. That study 51, however, neither showed images of growing organs nor assessed gene expression.

In future, it will be important to pinpoint the intracellular signalling mechanisms of TGFβ and in ureter development. This is a potentially complex field, with numerous pathways potentially involved 14. The canonical, TGFβ signalling pathway involves SMAD2 and SMAD3 that form complexes with SMAD4 which then move to the nucleus. Although we detected pSMAD2 on histology sections, this study did not quantify the signals. Moreover, another SMAD, SMAD7, inhibits the TGFβ pathway 14. Furthermore non‐canonical TGFβ signalling may be operative, too, involving p38 MAPK, JNK or NF‐κB 14. One way forward here would be to generate a series of mutant mice, each with a deletion of specific TGFβ receptors or SMADs or other intracellular signalling molecules in either the urothelium or the mesenchyme/SM or the adventitia. In this respect, it is notable that Mamo *et al* 41 used a *Tbx18Cre* driver to delete SMAD4 in differentiating SM of the ureter, noting only a modest delay in SM differentiation. In fact, TGFβ signalling is also thought to enhance SM differentiation in the developing intestine 30. The study of Mamo et al 41 would not have been informative with regard to potential TGFβ direct effects on the differentiating urothelium and this may explain why ectopic buds, prominent after TGFβ receptor inhibition in the current work, were not observed.

After 24 h of TGFβ1 exposure, the most up‐regulated transcripts in the RNA sequencing sets were those encoding: platelet‐derived growth factor‐like (*Pdgfrl*), implicated in chondrocyte differentiation 52; scleraxis (*Scx*), implicated in extracellular matrix molecule expression in tendons and heart 53; chondroadherin (*Chad*), which is enriched in cartilage 54; biglycan (*Bgn*), a matrix molecule that, like chondroadherin, is up‐regulated during TGFβ induced differentiation of mesenchymal stem cells towards cartilage 55; fibronectin‐1 (*Fn1*), a matrix protein implicated in myofibroblast formation 56; and matrix Gla protein (*Mgp*) that modulates urinary stone formation 57. These changes were consistent with increased adventitial prominence after TGFβ1 exposure, suggesting that its composition begins to shift towards a metaplastic cartilage‐like phenotype.

*Fgf18* was also up‐regulated in TGFβ1 exposed ureters. Rudiments also expressed *Fgfr3*, encoding the cell surface receptor for FGF18 58. Ours is the first report drawing attention to whether this ligand is expressed in the embryonic ureter, and exploring its potential relation to morphogenesis. When recombinant FGF18 was added to ureteric explants fed basal media alone, a small increase in linear growth occurred but exogenous FGF18 did not impact on TGFβ1‐induced dysmorphogesis. Further experiments are needed to determine whether endogenous *Fgf18* affects ureter development. FGF18 is related to FGF8 and FGF17 58 but *Fgf8* and *Fgf17* transcripts were barely detectable in ureter rudiments. *FGF18* polymorphisms are associated with facial clefting, *FGFR3* mutations cause skeletal dysplasias 35, and *Fgf18* deleted mice have delayed chondrocyte differentiation 59. Mice lacking FGF18 have impaired alveolar epithelial growth in development 60, and *Fgfr3* mutant mice have enhanced intestinal crypt proliferation 61. Other studies link FGF18 and TGFβ biology. In hair, TGFβ2 and FGF18 respectively accelerate and delay telogen‐to‐anagen transition in which new hair shafts are generated from stem cells 62. Finally, in bone cultures, TGFβ1 up‐regulates *Fgf18*, with increased FGFRIII phosphorylation 32.

Among down‐regulated transcripts were those encoding: sushi, nidogen and EGF‐like domains 1 (*Sned1*), an extracellular matrix protein found in embryonic kidneys 63 and implicated in tumour invasion 64; hippocalcin (*Hpca*), a calcium sensor implicated in neurodegeneration 65; solute carrier family 26A7 (*Slc26a7*), a Cl^−^/HCO_3_^−^ exchanger 66; solute carrier family 26A1 (*Slc4a1*), an anion exchanger 67; and tenascin XB (*Tnxb*). *TNXB* mutations cause human urinary tract malformations and healthy urothelia express tenascin XB 68, a protein that regulates TGFβ1 bioavailability 69. Robust levels of the following transcripts were detected at days 1 and 6 of culture in controls: *Shh*, *Ptch1*, *Smo*, *Gli1*, *Gli2* and *Bmp4*, encoding hedgehog pathway molecules that generate SM 70; and *Rara*, *Rarb*, *Rarg*, *Sox9*, *Tbx18* and *Tshz3*, encoding urinary tract transcription factors 3, 70, 71, 72, 73. Exogenous TGFβ1 did not alter these transcripts after correction for multiple comparisons. FGF10 is mitogenic for urothelia 33 and stimulates amniotic stem cells to acquire urothelial characteristics 74. We noted a tendency for down‐regulated *Fgf10* upon exposure to TGFβ1. When FGF10 was added to cultures, the growth inhibiting effect of TGFβ1 was abrogated. This introduces the idea that the dysmorphic effects of TGFβ1 can be modified by other growth factors expressed in the embryonic ureter. Notably, in an *ex vivo* model, exogenous TGFβ1 inhibited *Fgf10* expression in prostate‐related mesenchyme, an effect mediated through the *Fgf10* promoter 75.

Collectively, these observations reveal an unsuspected regulatory role for endogenous TGFβ signalling in embryonic ureters, fine‐tuning morphogenesis. The results also support the hypothesis that up‐regulation of TGFβ axis molecules plays roles in the pathobiology of ureter malformations. Further experiments are needed to unravel the intracellular signalling mechanisms involved in these dysmorphic responses.

## Author contributions statement

ASW, FML and NAG conceived and designed the study. FML, NAR and LAHZ undertook analyses. All authors contributed to data interpretation. ASW and FML drafted the paper. All authors approved the final version of the paper.

## Supporting information


**Supplementary materials and methods**

**Figure S1.** Immunodetection of TGFβRI and TGFβRII in a 10‐week human ureter
**Figure S2.** Immunodetection of TGFβRI and TGFβRII in the embryonic day 15 mouse ureter
**Figure S3.** RT‐qPCR of mouse ureters harvested between E13 and the day of birth
**Figure S4**. *In situ* hybridisation of embryonic day 15 mouse ureter
**Figure S5.** Gross morphology and growth of explanted E15 ureters
**Figure S6.** E13 ureter explants viewed as whole mounts in culture
**Figure S7.** Quantification of BrdU uptake 24 h after explanting embryonic day 15 ureters into culture
**Figure S8.** RNA‐sequencing data displayed as a volcano plot, comparing TGFβ1 exposed and control cultures at 24 h after being explanted
**Figure S9.** RT‐qPCR analyses of ureter explants
**Figure S10.** Effects of addition of FGF18 to mouse E15 ureters in organ culture
**Figure S11.** Immunostaining for FGF10
**Figure S12.** Effects of SB431542, a TGFβRI kinase inhibitor, on E15 ureter organ cultures
**Figure S13.** Effects of adding both TGFβ1 and a TGFβ receptor blocker

**Table S1.** Transcripts encoding TGFβ family members and their receptors in explanted embryonic ureters
**Table S2.** The most significantly changed transcripts in TGFβ1 exposed ureters versus control ureters on day 1 of E15 organ culture
**Table S3.** Transcripts encoding FGF family members and their receptors, as detected and quantified in the RNA‐sequencing analyses
