# Supplementary material for: Overactivity or blockade of transforming growth factor‐β each generate a specific ureter malformation
Source: J Pathol. 2019 Oct 1;249(4):472–84. doi: 10.1002/path.5335 (PMC6900140; doi:10.1002/path.5335)
Supplement: Supplementary file 1 — Supplementary materials and methods [file PATH-249-472-s003.docx]

**Overactivity or blockade of transforming growth factor-β each generate a specific ureter malformation**

Lopes FM *et al. J Pathol* DOI: 10.1002/path.5335

**Supplementary materials and methods**

**Histology**

Tissues were fixed in 4% paraformaldehyde and sectioned at 5 μm thickness. After dewaxing and rehydration, endogenous peroxidase was quenched by incubation with hydrogen peroxide. Brightfield immunohistochemistry was undertaken with primary antibodies: mouse anti-αSM actin (αSMA; 1:8000, A2547, Sigma-Aldrich, Gillingham, UK); mouse anti-bromodeoxyuridine (BrdU; 1:200, sc-51514, Santa Cruz Biotechnology, Heidelberg, Baden-Württemberg, Germany)  mouse anti-cadherin-1 (CDH1, also known as E-cadherin; 1:600 for human and mouse tissues; ab76055, Abcam, Cambridge, UK); rabbit anti-FGF10 (1:50; ABN44; Sigma-Aldrich); rabbit anti-pSMAD2 Ser465, Ser467 (1:400, 44-244G, Thermo Fisher Scientific, Paisley, UK); rabbit anti-TGFβRI (1:100 for human tissue and 1:400 for mouse tissue; ab31013, Abcam); rabbit anti-TGFβRII (1:200 for human tissue and 1:400 for mouse tissue; ab186838, Abcam); and goat anti-uroplakin II (UPKII, 1:600 for human and mouse tissues; sc-15178, Santa Cruz Biotechnology). Before applying primary antibodies, slides were microwaved for 5 min and then cooled at room temperature for 20 min in antigen-retrieval solution (10 mM sodium citrate, pH 6.0). Before using primary antibodies raised in mice, mouse endogenous Ig staining was abrogated using Elite Peroxidase (BMK-2200; Vector Laboratories, Peterborough, UK). Each antibody was mixed with PBS Triton-X (0.1%) and 3% serum specific to the species of the secondary antibody and incubated overnight at 4 °C. Primary antibodies were detected using appropriate secondary antibodies, detected with a DAB peroxidase-based method. In some sections, picrosirius red staining [26] was used to identify collagens, and some sections were counterstained with haematoxylin or haematoxylin and eosin. Negative controls consisted of omission of primary antibodies. Images were collected on an Olympus BX63 upright microscope using a DP80 camera (Olympus) through CellSens Dimension software v1.16 (Olympus, Southend-on-Sea, UK). For immunofluorescence studies, slides were deparaffinized and rehydrated, followed by antigen retrieval in 10 mM sodium citrate, pH 6.0 for 5 min in a microwave. Sections were then blocked with serum specific for the secondary antibody species. Primary antibodies used were rabbit anti-TGFβRI (1:200; ab31013, Abcam) and rabbit anti-TGFβRII (1:1000; ab186838, Abcam) were diluted in 1% BSA and PBS in 0.1% Tween 20 and incubated overnight at 4 °C. Fluorophore-conjugated secondary antibody was incubated for 1 h at room temperature, and DAPI was used to counterstain nuclei. Images were acquired on an Olympus IX83 inverted microscope and deconvolved using Scientific Volume Imaging software (Huygens Professional, Hilversum, The Netherlands).

***In situ* hybridisation.**

Embryos were fixed in 4% paraformaldehyde treated with diethyl pyrocarbonate and sectioned at 5 μm. RNAscope® Assays (ACDBio, Newark, CA, USA) were undertaken according to the manufacturer’s instructions using the RNAscope® 2.5 LS Reagent Kit RED (ACDBio) and the BOND RX System (Leica Biosystems, Milton Keynes, UK). RNA detection (red) and nuclear counterstaining (blue) were undertaken using Leica BOND Red Detection plus Haematoxylin kit. Images were acquired using an Olympus BX63 upright microscope using a DP80 camera (Olympus) through CellSens Dimension v1.16 software (Olympus). The following RNAscope® 2.5 LS probes were used: Mm-*Tgfb1* 407758; Mm-*Tgfbr1* 406208; Mm-*Tgfbr2* 406248; Mm-*Ppib* 313918 (positive control); and *dapB* 312038 (negative control). The positive control probe was the widely expressed *Ppib* transcript encoding peptidylprolyl isomerase B, and a negative control probe was *DapB* encoding 4-hydroxy-tetrahydrodipicolinate reductase from the *Bacillus subtilis* soil bacterium, a gene that is absent in mammals.

**RNA sequencing.**

Organs were collected in RNA protect (Thermo Fisher Scientific) and RNA extracted using RNeasy Plus Mini Kit (Qiagen, Manchester UK). Libraries were generated with TruSeq Stranded mRNA Library Prep Kit and sequenced paired-end on the Illumina HiSeq4000 platform. Unmapped paired-end sequences from an Illumina HiSeq4000 sequencer were tested by FastQC (<http://www.bioinformatics.babraham.ac.uk/projects/fastqc/>). Adapter sequences were removed and reads were quality trimmed using Trimmomatic_0.36 [23]. Reads were mapped against the reference genome (mm10/GRCm38) and counts/gene calculated by annotation from GENCODE M14 (<http://www.gencodegenes.org/>) using STAR_2.5.3 [24]. Normalisation, Principal Components Analysis, and differential expression were calculated using DESeq2_1.16.1 [25]. Differentially expressed transcripts were defined as those showing a fold-change increase of at least 1.5. or decrease of at least 0.67, and a statistical significance of p<0.05 after correcting for multiple comparisons. Each n=1 analysis comprised RNA extracted from six to eight ureters grown in one condition for one or six days. Comparisons (n=3 for each experimental set) were made with paired ureter sets from the same litters. Data was deposited in the ArrayExpress repository (E-MTAB-7395).

**RT-qPCR**

RNA was extracted as described in the RNA sequencing method, and one μg used to generate cDNA using the High-Capacity RNA-to-cDNA kit (Thermo Fisher Scientific). cDNA was diluted to a 1/20 working concentration and 10 μl reactions were performed using one μl of cDNA working stock. Reactions were performed using Taqman Gene Expression MasterMix (Thermo Fisher Scientific) on a StepOnePlus platform (Thermo Fisher Scientific) and data analyzed using StepOne software and rendered using Graphpad PRISM (Graphpad Software Inc, San Diego, CA, USA). Taqman assay IDs used were: *Bgn* Mm01191753_m1l; *Fgf10* Mm00433275_m1; *Fgf18* Mm00433286_m1; *Hprt* Mm03024075_m1; *Pdgfrl* Mm00452798_m1; *Sox9* Mm00448840_m1; *Tgfb1* Mm01178820_m1; *Tgfb2* Mm00436955_m1; Tgfb3 Mm00436960_m1; *Tgfbr1* Mm00436964_m1; *Tgfbr2* Mm03024091_m1; and *Upk3a* Mm00452321_m1.
