# Supplementary material for: Overactivity or blockade of transforming growth factor‐β each generate a specific ureter malformation
Source: J Pathol. 2019 Oct 1;249(4):472–84. doi: 10.1002/path.5335 (PMC6900140; doi:10.1002/path.5335)
Supplement: Supplementary file 2 — Figure S1. Immunodetection of TGFβRI and TGFβRII in a 10‐week human ureter Figure S2. Immunodetection of TGFβRI and TGFβRII in the embryonic day 15 mouse ureter Figure S3. RT‐qPCR of mouse ureters harvested between E13 and the day of birth Figure S4. In situ hybridisation of embryonic day 15 mouse ureter Figure S5. Gross morphology and growth of explanted E15 ureters Figure S6. E13 ureter explants viewed as whole mounts in culture Figure S7. Quantification of BrdU uptake 24 h after explanting embryonic day 15 ureters into culture Figure S8. RNA‐sequencing data displayed as a volcano plot, comparing TGFβ1 exposed and control cultures at 24 h after being explanted Figure S9. RT‐qPCR analyses of ureter explants Figure S10. Effects of addition of FGF18 to mouse E15 ureters in organ culture Figure S11. Immunostaining for FGF10 Figure S12. Effects of SB431542, a TGFβRI kinase inhibitor, on E15 ureter organ cultures Figure S13. Effects of adding both TGFβ1 and a TGFβ receptor blocker [file PATH-249-472-s001.docx]

**Overactivity or blockade of transforming growth factor-β each generate a specific ureter malformation**

Lopes FM *et al. J Pathol* DOI: 10.1002/path.5335


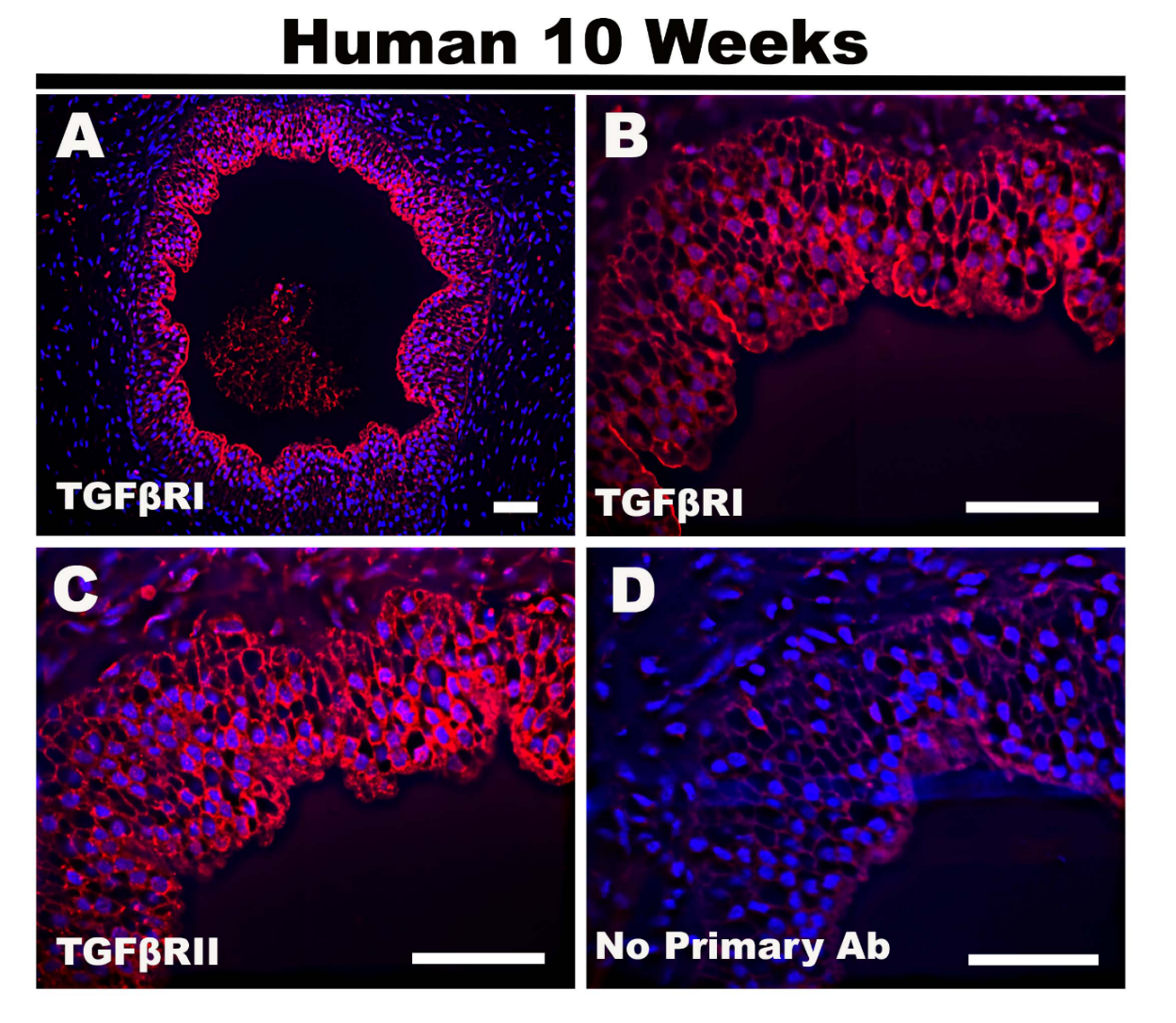


**Figure S1. Immunodetection of TGFβRI and TGFβRII in a ten-week human ureter.** Transverse section near the top of the ureter as it connects with the renal pelvis. Fluorescence immunohistochemistry detected a plasma membrane-like localisation of TGFβRI (red colour; low power, A; high power, B) and TGFβRII (high power, C) in the multi-layered urothelium. Note the minimal faint background signal when the primary antibody was omitted (D). Nuclei were counterstained with DAPI (blue). Bars, 20 μm.


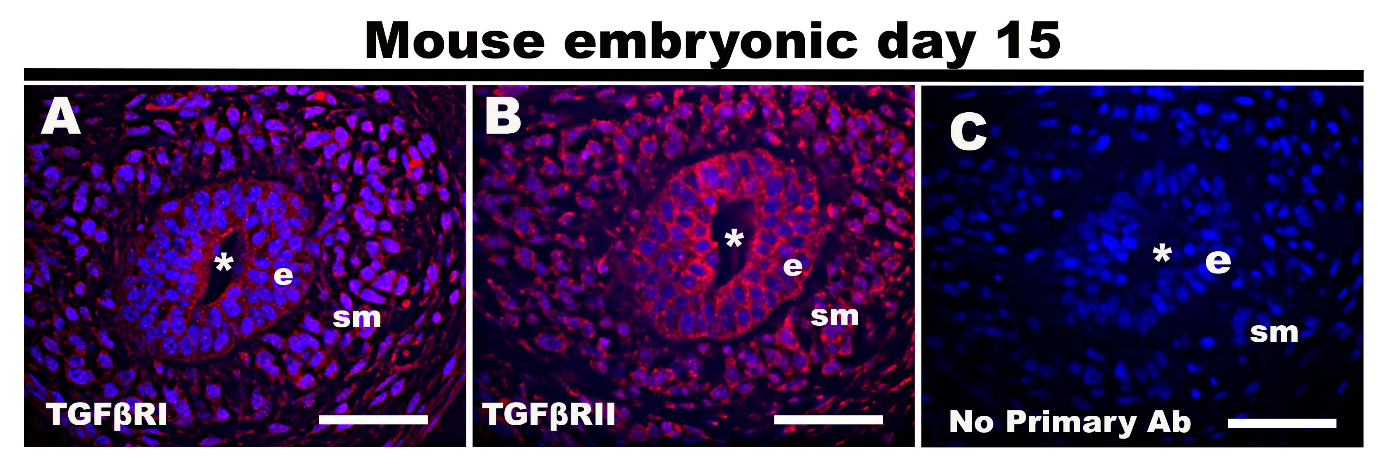


**Figure S2. Immunodetection of TGFβRI and TGFβRII in the embryonic day 15 mouse ureter.** Fluorescence immunohistochemistry detected (red) (A) TGFβRI, and (B) TGFβRII in the urothelium (e) and surrounding nascent smooth muscle (sm). Note there was no background signal when the primary antibody was omitted (C). The lumen is indicated by the asterisk. Bars, 20 μm.

**
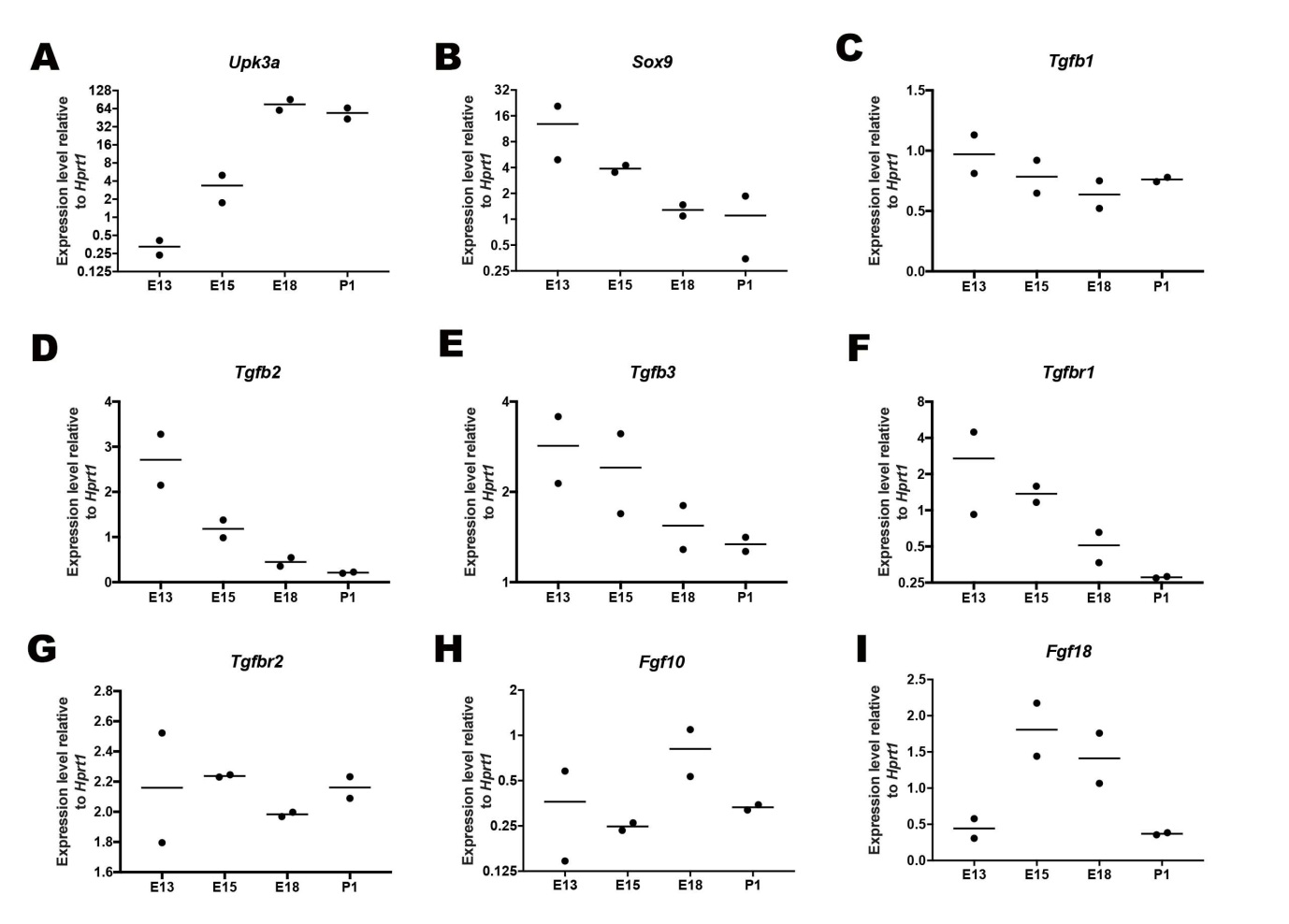
**

**Figure S3. RT-qPCR of mouse ureters harvested between E13 and the day of birth.** RT-qPCR for *Tgfb1*, *Tgfb2*, *Tgfb3*, *Tgfbr1* and *Tgfbr2* using mRNA from mouse ureters, with target transcripts normalised to the housekeeping transcript *Hprt1*. Transcripts encoding TGFβ1, TGFβ2 and TGFβ3 ligands, as well as the two key TGFβ receptors for these ligands, are all expressed over a wide span of mouse ureter development i.e. at embryonic days 13, 15 and 18 (E13, E15, and E18), and on the day of birth (P1). As expected, over this period, transcripts for *Upk3a*, encoding the epithelial maturation marker uroplakin 3A, tended to rise, while transcripts for *Sox9*, encoding a transcription factor that regulates ureteric SM differentiation, tended to fall. *Fgf10* and *Fgf18* transcripts were also detected at all time points. Each point represents RNA extracted from a pool of time-matched ureters


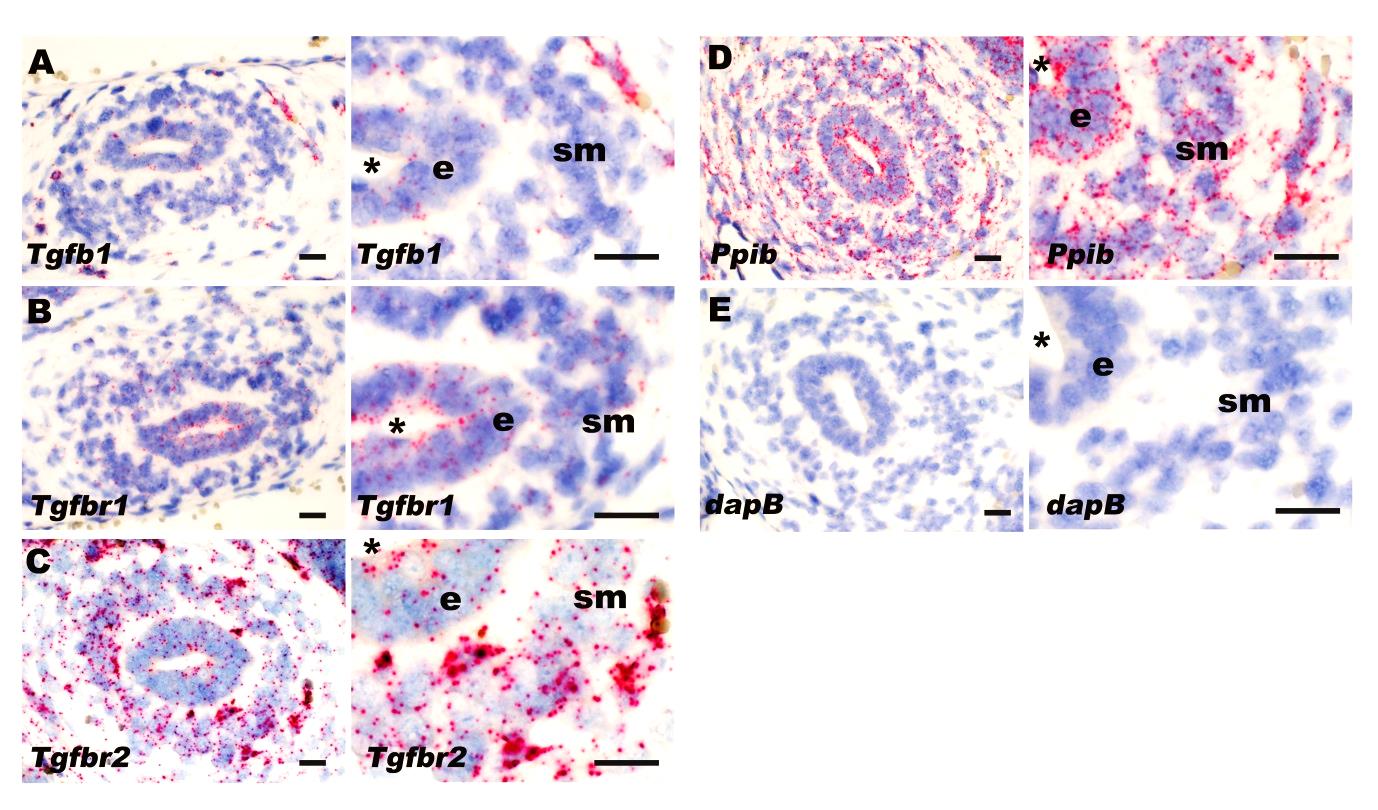


**Figure S4. *In situ* hybridisation of embryonic day 15 mouse ureter.** RNAScope^®^ *in situ* hybridisation was performed on transverse histological sections of embryonic day 15 mouse ureters to seek *Tgfb1*, *Tgfbr1* and *Tgfbr2* mRNAs. RNA signals appear as red dots; nuclei were counterstained blue with haematoxylin. For each transcript, the left-hand image gives an overview, and the right-hand frame an enlargement. Relatively few signals for (A) *Tgfb1*were detected in the epithelium (e). More prominent signals were detected for (B) *Tgfbr1* and (C) *Tgfbr2*. Both transcripts were detected in the epithelium. Prominent *Tgfbr2 e*xpression was also detected in smooth muscle (sm), in laminal propria cells between the muscle and epithelium, and in adventitia outside the smooth muscle*.* (D) *Ppib* transcripts encoding peptidylprolyl isomerase B were used as a positive control and the negative control probe was (E) *DapB* encoding 4-hydroxy-tetrahydrodipicolinate reductase, a gene that is absent in mammals: note the absence of red dots. Size bars, 20 μm

**
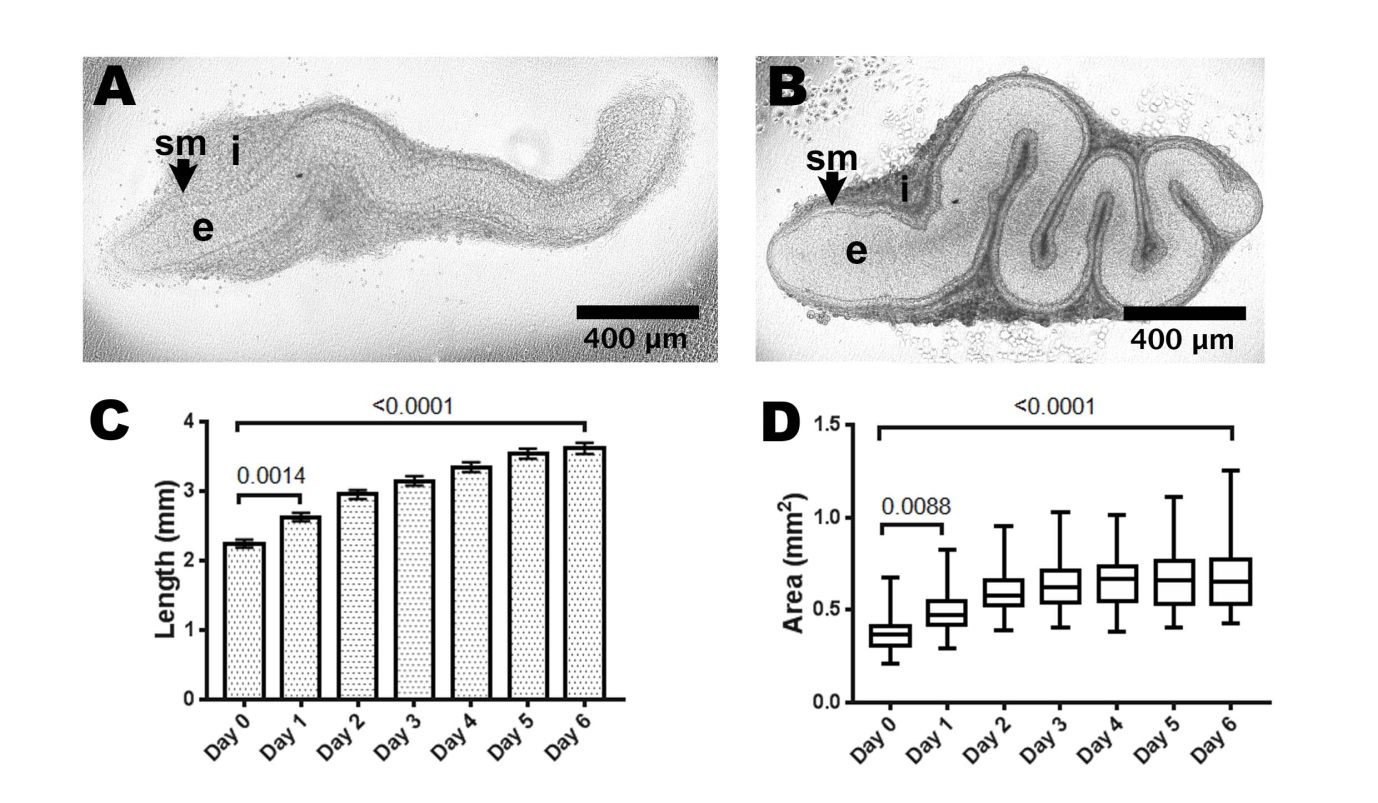
**

**Figure S5. Gross morphology and growth of explanted E15 ureters.** (A) On the day of being explanted, visualised as a whole mount, the E15 ureter (proximal end at the left of frame) had an epithelial central zone (*e*) surrounded by a thin lighter zone that represents the nascent SM layer (sm, arrowed) that itself was surrounded by loose interstitial cells (i). (B) After six days in culture, the same organ had elongated and acquired a snake-like shape. The three layers were still apparent, with the SM layer having become more defined. (C) Daily measurements (mean ± SEM, n=51) of the length of the ureter tube in organ culture. After one day, the length significantly increased (p=0.014) and by day six, the end of culture period, rudiments had elongated an average of 1.2 mm. (D) Daily measurements (median, interquartile range and range, n=51) of the combined area of epithelial and SM compartments. Note that, in these culture conditions, increases of length and area have plateaued by day six.


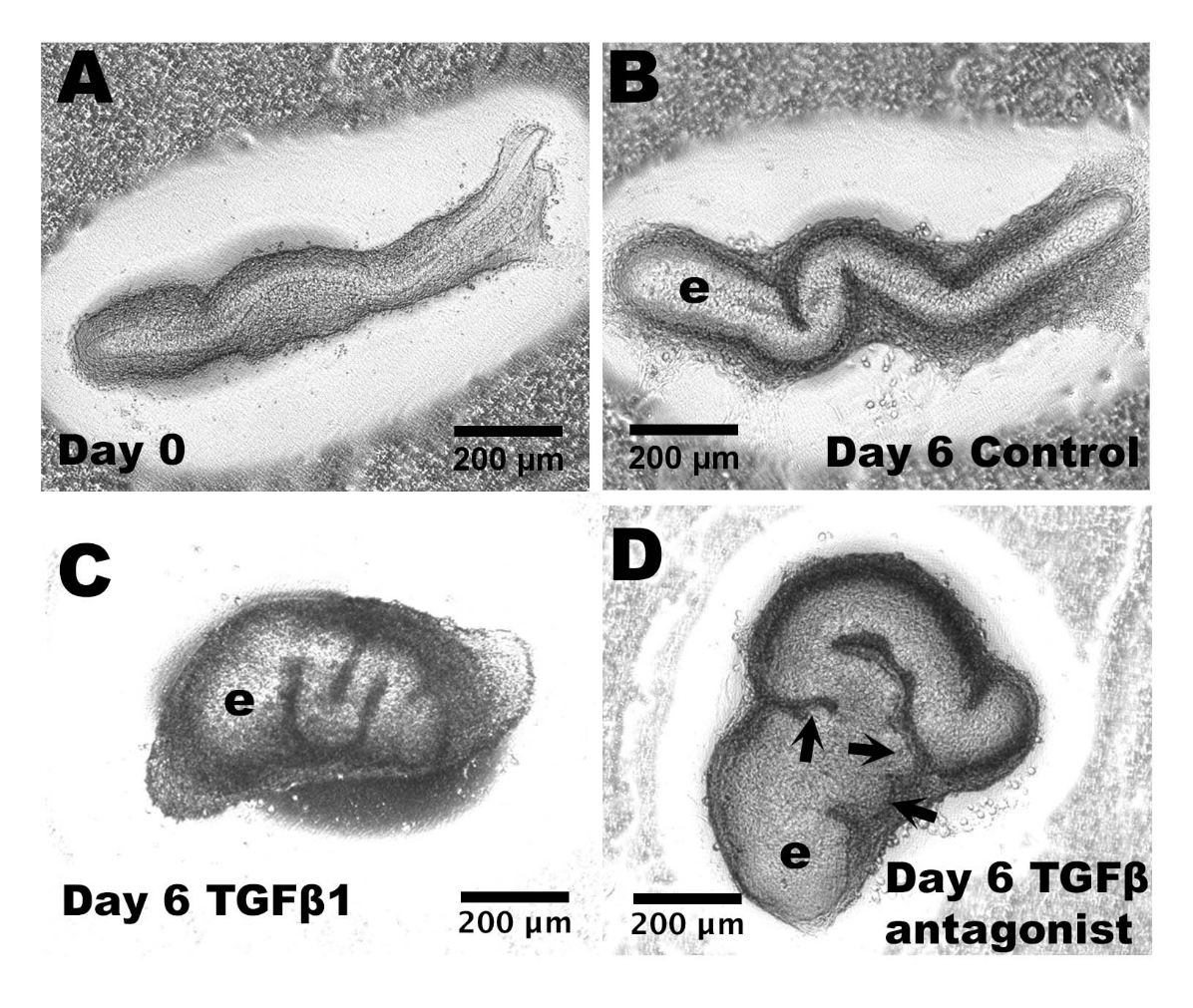


**Figure S6. E13 ureter explants viewed as whole mounts in culture.** (A) E13 rudiment on the day of being explanted. (B) The same E13 rudiment fed basal media alone for six days. (C) An E13 rudiment after six days of culture fed with media supplemented with 5 ng/ml TGFβ1. Note the cocoon-like appearance. (D) E13 rudiment after six days of culture fed with media supplemented with 10 μM LY2109761, a molecule that inhibits TGFβRI/TGFβRII kinase. Note the overgrown appearance with several bud-like structures (*arrows*). The epithelial zone is indicated by e. Images are representative of six explants in each condition.

**
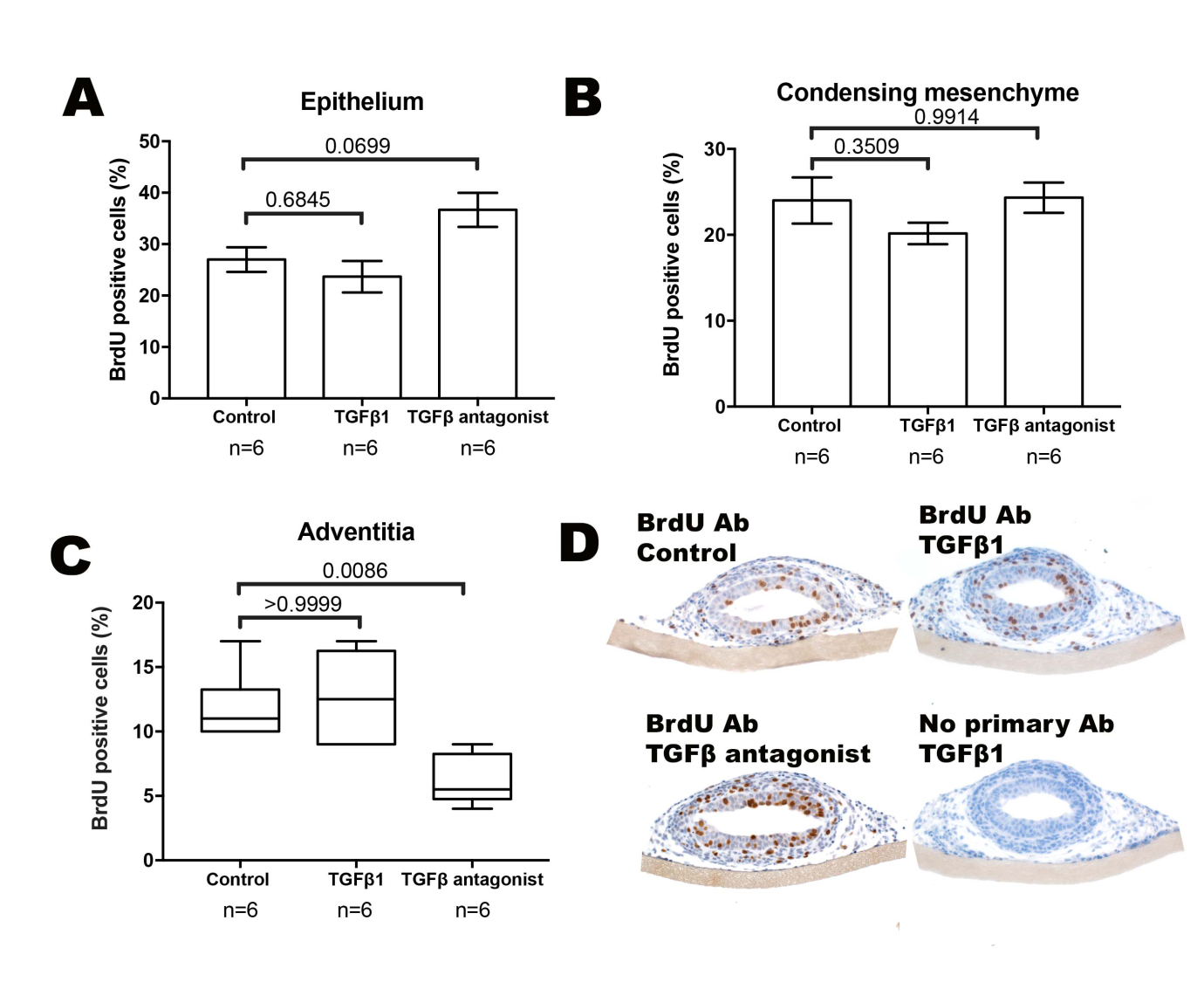
**

**Figure S7. Quantification of BrdU uptake 24 hours after explanting embryonic day 15 ureters into culture.** A–C show proportions of nuclei that had incorporated BrdU. While there were no changes after exposure to 5 ng/ml TGFβ1, exposure to 10 μM LY2109761, a molecule that inhibits TGFβRI/TGFβRII kinase (TGFβ1 antagonist), was associated with a tendency for increased proliferation in the epithelium and a significantly decreased proliferation in the adventitia. (D) representative immunohistochemical staining (*brown nuclei*) for BrdU in each of the three conditions, with the lower right panel being a section with the anti-BrdU antibody omitted. For each group, n=6. For parametric data, results are means and SEMs, while for non-parametric data results are medians, interquartile ranges, and absolute range


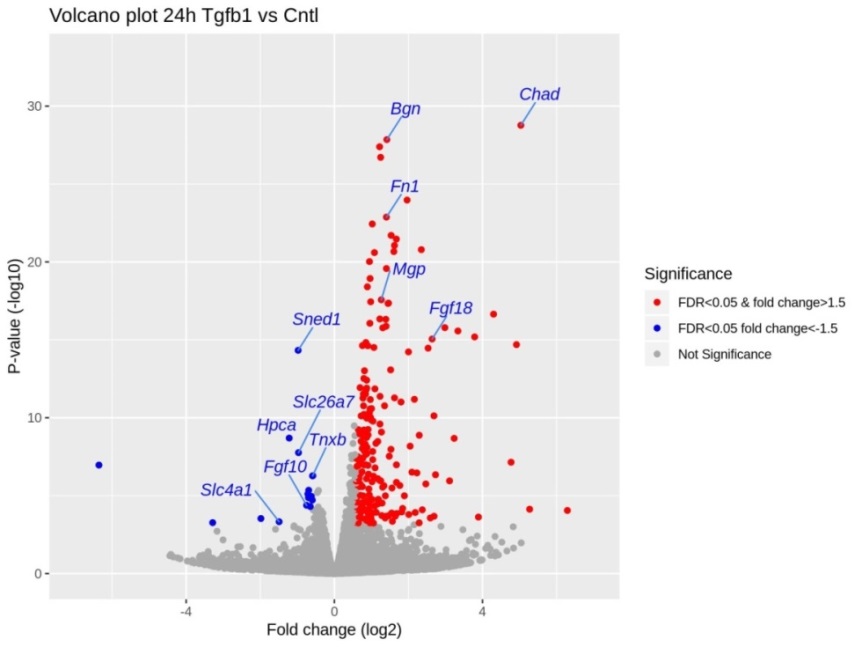


**Figure S8. RNA-sequencing data displayed as a volcano plot, comparing TGFβ1-exposed and control cultures at 24 hours after being explanted.** After adjusting for multiple comparisons, significantly upregulated transcripts are shown in red, and downregulated transcripts in blue. The annotated transcripts are those considered in the *Results* and *Discussion*. The horizontal axis is the fold-change using a log2 scale of TGFβ1-exposed cultures (n=3) compared with control cultures (n=3). The vertical axis is the p value adjusted for multiple comparisons on a log10 scale.

**
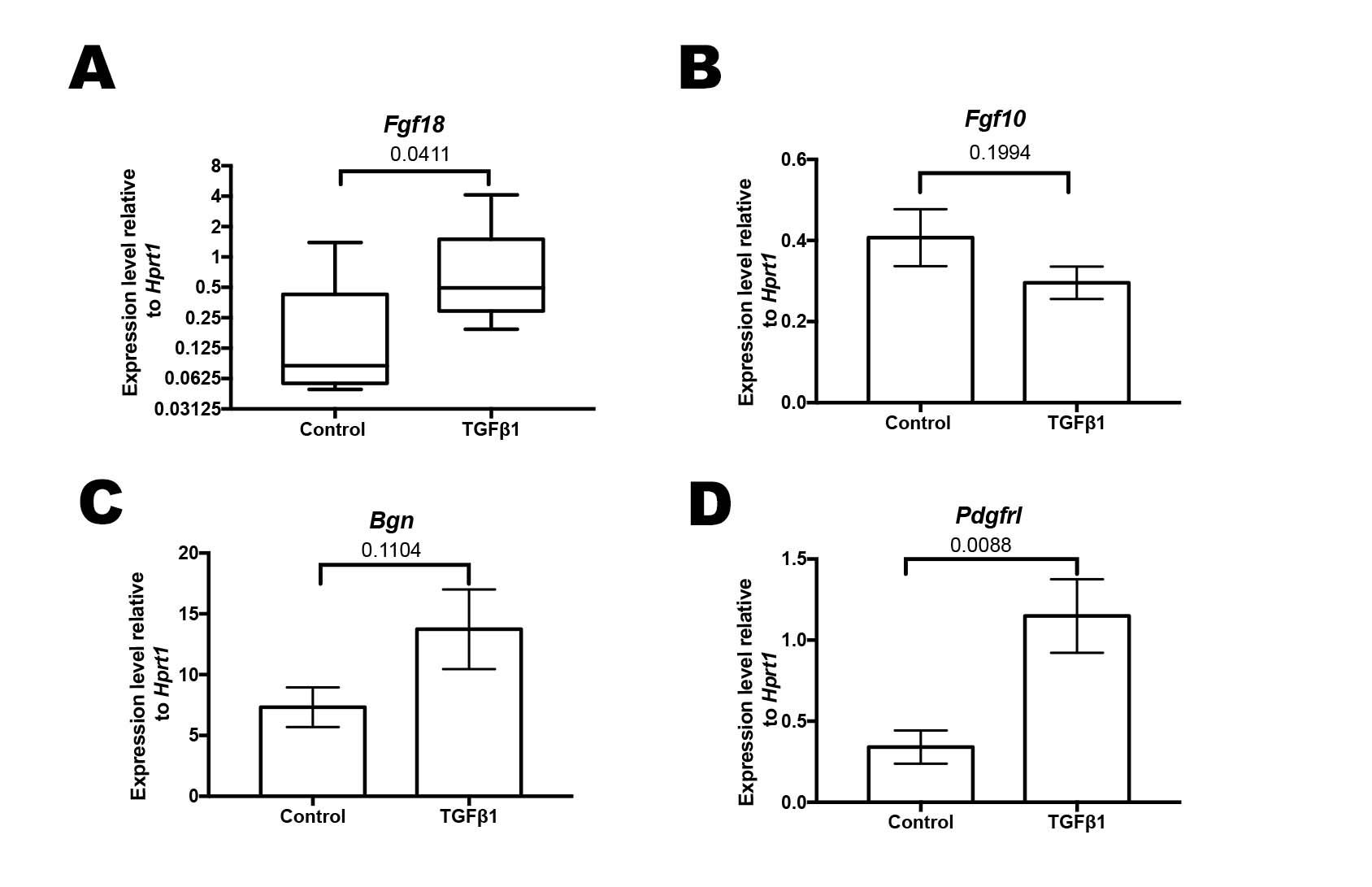
**

**Figure S9. RT-qPCR analyses of ureter explants.** Embryonic day 15 ureter explants were harvested after one day in culture. For each group, n=6, with 10 ureters pooled to generate each sample. For parametric data, results are means and SEMs, while for non-parametric data results are medians, interquartile ranges, and absolute ranges.

**
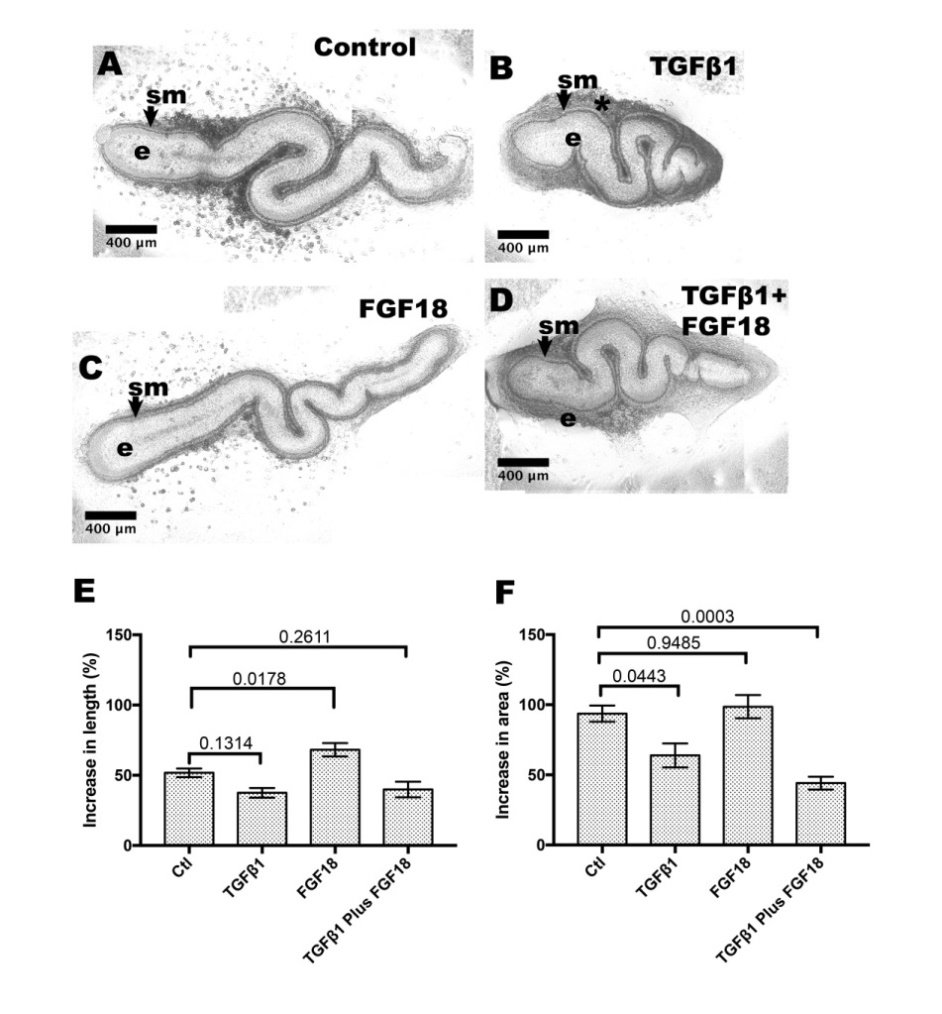
**

**Figure S10. Effects of addition of FGF18 to mouse E15 ureters.** (A-D) Explants viewed at day six. (A) Organ fed basal media only. SM (sm, arrowed) and epithelial (e) zones are indicated. (B) Organ fed with media supplemented with 5 ng/ml TGFβ1. Note cocoon with ureter tube surrounded by a dense interstitial layer (***). (C) Organ fed with media supplemented with 200 ng/ml FGF18. Note elongated appearance versus basal media alone. (D) Organ fed with basal media supplemented with both 5 ng/ml TGFβ1 and 200 ng/ml FGF18. TGFβ1-induced cocooning was still apparent, with prominent interstitial tissue (***). (E,F) Quantification of percent-increases, compared with values on the day of being explanted, of (E) lengths and (F) areas of ureter tubes. FGF18 caused a small statistically-significant increase in linear growth versus basal media. When FGF18 was added with TGFβ1, the negative effect of the latter on area growth was still apparent. Data are mean ± SEM, n=8 in all groups.


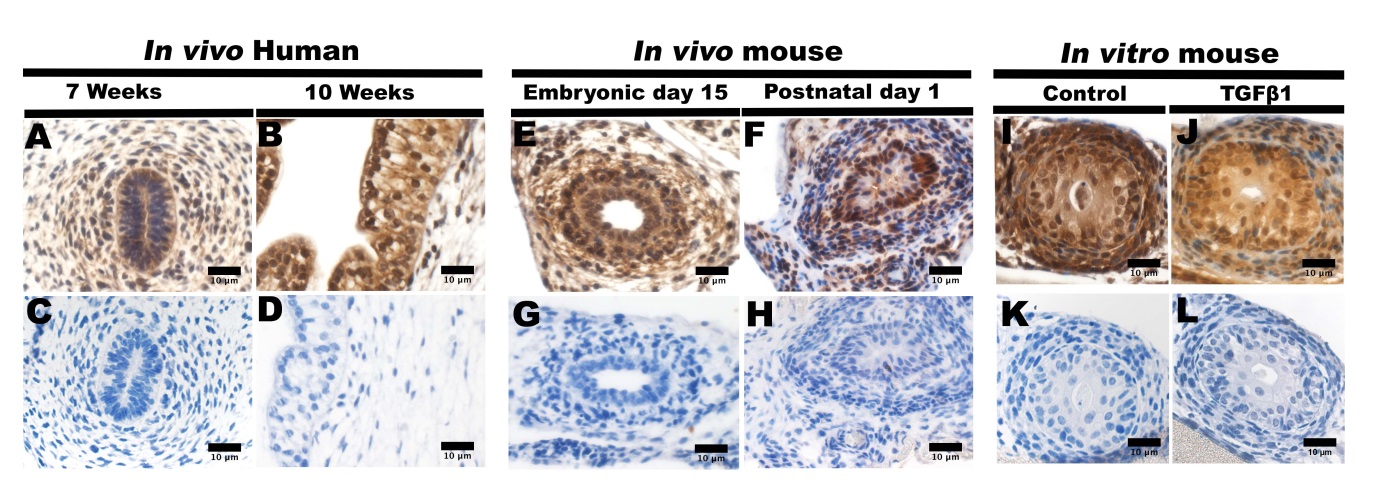


**Figure S11. Immunostaining for FGF10.** FGF10 was immunodetected in (A,B) human embryonic ureters *in vivo* at seven and ten weeks gestation, (E,F) in mouse embryonic ureters *in vivo* at E15 and neonatally, and on day six of mouse E15 ureter culture where the signal appeared stronger in (I) control than in (J) TGFβ1-exposed explants. (C–L) Nearby sections in which the primary antibody was not applied. Bars, 10 μm.

**
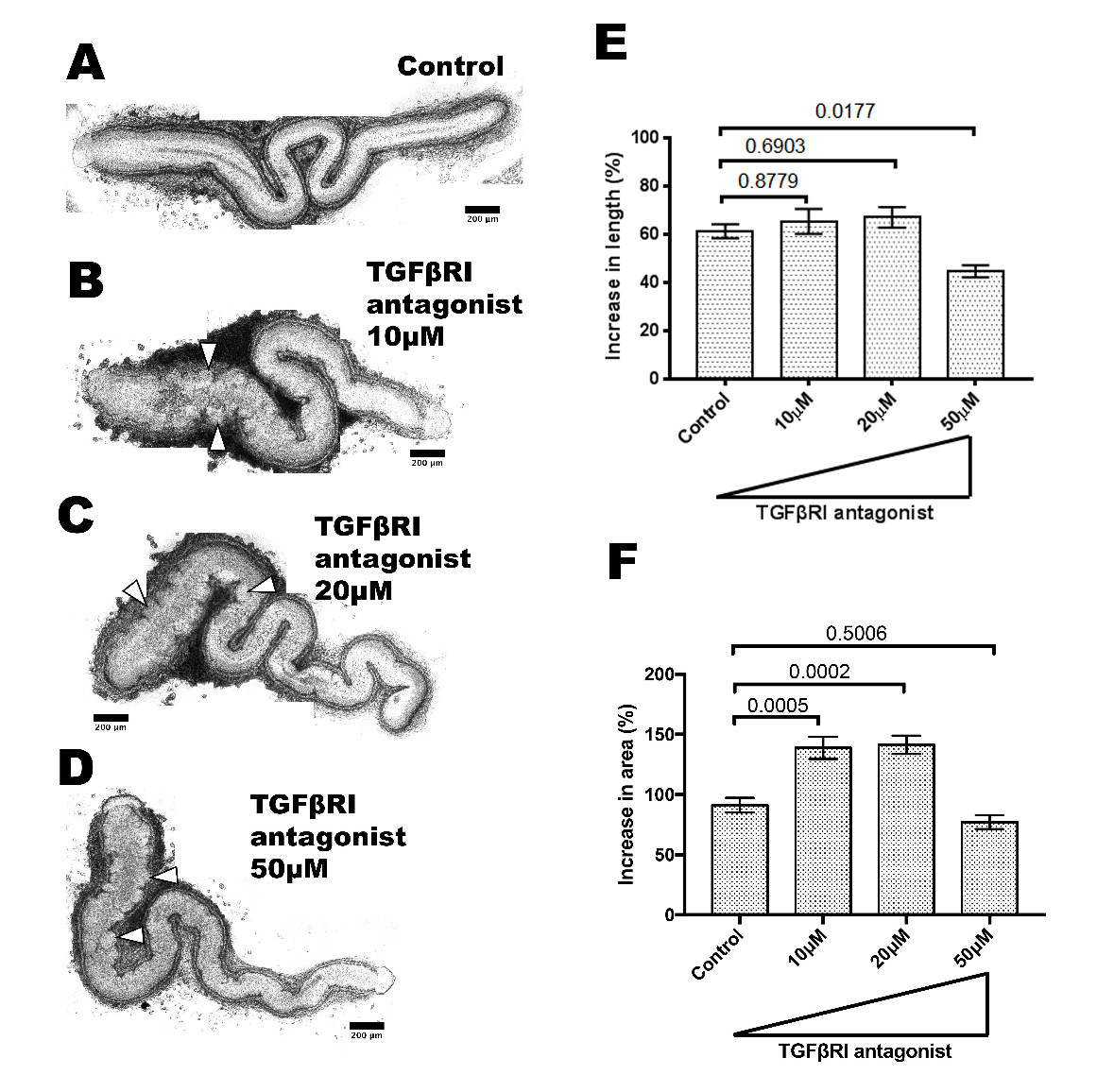
**

**Figure S12. Effects of SB431542, a TGFβRI kinase inhibitor, on E15 ureter organ cultures.** (A–D) Explants viewed from above as whole mounts at day six of culture. (A) An organ fed basal media only. (B,C) Explants supplemented with 10, 20 or 50 μM SB431542. Note that bud-like structures (*white arrowheads*) were generated by all three concentrations, most prominently in the proximal part of the ureter tube. (E,F) Quantification of increases, compared with values on the day of being explanted, of (E) length and (F) area of ureter tubes. Note the significant increase in explant area growth produced by 10 and 20 μM SB431542. In the highest concentration, 50 μM, the area was not different from controls, yet the budding effect still occurred. Data are mean ± SEM, n=10 in control group, 7 in the 10 μM SB431542 group, 8 in the 20 μM SB431542 group and 8 in the 50 μM SB431542 group.

**

**

**Figure S13. Effects of adding both TGFβ1 and a TGFβ receptor blocker.** A-D. Representative images of embryonic day 15 ureters explanted and analysed after six days. Size bars, 200 μm. Compared with the control (A), note the expected ‘cocooning’ upon exposure to 5 ng/ml TGFβ1, and apparent elongation upon exposure to 10 μM LY2109761 (C). Cultures simultaneously exposed to both agents (D) appeared to show less cocooning. Quantification of (E) length and (F) area was undertaken (n-5 to 6 organs per group). Analyses by one-way ANOVA (parametric) followed by multiple test comparisons showed that this inhibition of area growth elicited by TGFβ1 alone was abolished when the receptor blocker was applied at the same time.
