# Supplementary material for: Overactivity or blockade of transforming growth factor‐β each generate a specific ureter malformation
Source: J Pathol. 2019 Oct 1;249(4):472–84. doi: 10.1002/path.5335 (PMC6900140; doi:10.1002/path.5335)
Supplement: Supplementary file 3 — Table S1. Transcripts encoding TGFβ family members and their receptors in explanted embryonic ureters Table S2. The most significantly changed transcripts in TGFβ1 exposed ureters versus control ureters on day 1 of E15 organ culture Table S3. Transcripts encoding FGF family members and their receptors, as detected and quantified in the RNA‐sequencing analyses [file PATH-249-472-s002.docx]

**Overactivity or blockade of transforming growth factor-β each generate a specific ureter malformation**

Lopes FM *et al. J Pathol* DOI: 10.1002/path.5335

**Table S1. Transcripts encoding TGFβ family members and their receptors in explanted embryonic ureters**

| **RNA**  **name** | **Encoded molecule** | **Control**  **day one** | **Control**  **day six** | **TGFβ1**  **day one** | **TGFβ1**  **day six** |
| --- | --- | --- | --- | --- | --- |
| **TGFβ ligands** | | | | | |
| *Tgfb1* | Transforming growth factor 1 | 1199 | 3134**^a^** | 1394 | 2191 |
| *Tgfb2* | Transforming growth factor 2 | 2819 | 1365 | 2710 | 1842 |
| *Tgfb3* | Transforming growth factor 3 | 4016 | 2394 | 6807**^b^** | 4544 |
| **TGFβ receptors** | | | | | |
| *TgfbrI* | Transforming growth factor receptor I | 4585 | 3006 | 4864 | 3932 |
| *TgfbrII* | Transforming growth factor receptor II | 6957 | 5784 | 7584 | 8030 |
| *TgfbrIII* | Transforming growth factor receptor III | 3983 | 3390 | 2859**^c^** | 3638 |

The table shows the mean numbers of reads for each transcript at day one and six of culture in the control (i.e. fed basal media only) ureter group (n=3) and groups of organs (n=3) exposed to exogenous TGFβ1 (5 ng/mL). ^a^Control day one versus control day six p=0.0034; ^b^Control day one versus TGFβ1 day one p=1.56E-09; and ^c^Control day one versus TGFβ1 day one p=0.0261.

**Table S2. The most significantly changed transcripts in TGFβ1 exposed ureters versus control ureters on day one of E15 organ culture.**

| **RNA**  **name** | **Encoded molecule** | **TGFβ1** | **Control** | **Log2 fold change** | **p value** |
| --- | --- | --- | --- | --- | --- |
| **Up-regulated transcripts** | | | | | |
| *Pdgfrl* | platelet-derived growth factor  receptor-like | 1391 | 212 | 2.680 | 1.59E-67 |
| *C1qtnf3* | C1q and tumour necrosis factor  related protein 3 | 705 | 31 | 4.428 | 1.18E-58 |
| *Adamtsl2* | ADAMTS-like 2 | 681 | 25 | 4.713 | 9.22E-57 |
| *Scube1* | signal peptide | 2778 | 474 | 2.567 | 1.55E-40 |
| *Dclk1* | doublecortin like kinase 1 | 4234 | 1205 | 1.803 | 8.64E-39 |
| *Gxylt2* | glycoside xylosyltransferase 2 | 2935 | 957 | 1.612 | 2.93E-35 |
| *Pmepa1* | prostate transmembrane protein | 5957 | 2361 | 1.324 | 3.83E-35 |
| *Nfatc1* | nuclear factor of activated T cells | 1988 | 710 | 1.480 | 3.30E-33 |
| *Scx* | scleraxis | 370 | 52 | 2.807 | 1.43E-31 |
| *Chad* | chondroadherin | 219 | 6 | 5.034 | 5.04E-26 |
| *Bgn* | biglycan | 41528 | 15471 | 1.417 | 3.78E-25 |
| *Mfap4* | microfibrillar-associated protein 4 | 20588 | 8846 | 1.222 | 1.00E-24 |
| *Rgs5* | regulator of G-protein signalling 5 | 8135 | 3459 | 1.250 | 4.40E-24 |
| *Ptprv* | protein tyrosine phosphatase | 610 | 158 | 1.964 | 2.26E-21 |
| *Fn1* | fibronectin 1 | 62424 | 22927 | 1.406 | 2.63E-20 |
| *Sulf2* | sulfatase 2 | 14459 | 7093 | 1.024 | 6.76E-20 |
| *Pi16* | peptidase inhibitor 16 | 671 | 236 | 1.533 | 3.45E-19 |
| *Pcdh8* | protocadherin 8 | 584 | 180 | 1.673 | 5.59E-19 |
| *Nuak1* | NUAK family 1 | 951 | 299 | 1.624 | 1.36E-18 |
| *Spsb1* | splA/ryanodine receptor domain  and SOCS box containing 1 | 879 | 166 | 2.349 | 2.42E-18 |
| *Plxna4* | plexin A4 | 1257 | 409 | 1.607 | 3.04E-18 |
| *Nrk* | nik related kinase | 7679 | 3564 | 1.082 | 3.37E-18 |
| *Fam210b* | family with sequence similarity 210 | 2071 | 1075 | 0.948 | 1.22E-17 |
| *Mfap5* | microfibrillar associated protein 5 | 1547 | 586 | 1.406 | 3.26E-17 |
| *Egfl6* | EGF-like-domain | 2070 | 1057 | 0.965 | 1.37E-16 |
| *Mfap2* | microfibrillar-associated protein 2 | 7686 | 4153 | 0.890 | 4.49E-16 |
| *Mgp* | matrix Gla protein | 5039 | 2174 | 1.266 | 2.94E-15 |
| *Gas2* | growth arrest specific 2 | 1218 | 616 | 0.981 | 3.82E-15 |
| *Cmklr1* | chemokine-like receptor 1 | 445 | 163 | 1.462 | 4.49E-15 |
| *Megf6* | multiple EGF-like-domains 6 | 922 | 338 | 1.449 | 4.56E-15 |

**Table S2 (continued)**

| **Down-regulated transcripts** | | | | | |
| --- | --- | --- | --- | --- | --- |
| *Sned1* | sushi, nidogen and EGF-like domains 1 | 4117 | 8114 | -0.97772 | 2.97E-12 |
| *Hpca* | hippocalcin | 177 | 425 | -1.2186 | 6.43E-07 |
| *Slc26a7* | solute carrier family 26 | 1060 | 2065 | -0.96783 | 4.77E-06 |
| *Alb* | albumin | 0 | 43 | -6.35562 | 2.55E-05 |
| *Tnxb* | tenascin XB | 2101 | 3156 | -0.58846 | 0.000108 |
| *Efemp1* | epidermal growth factor-containing  fibulin-like extracellular matrix protein 1 | 614 | 987 | -0.69322 | 0.000723 |
| *Arhgap20* | Rho GTPase activating protein 20 | 456 | 734 | -0.71565 | 0.001229 |
| *Enpp2* | ectonucleotide pyrophosphatase/  phosphodiesterase 2 | 3495 | 5271 | -0.61896 | 0.001675 |
| *Ebf1* | early B cell factor 1 | 1156 | 1936 | -0.70018 | 0.002011 |
| *Gbp9* | guanylate-binding protein 9 | 370 | 560 | -0.59409 | 0.002729 |
| *Fgf10* | fibroblast growth factor 10 | 602 | 1069 | -0.75013 | 0.005216 |
| *Aox3* | aldehyde oxidase 3 | 283 | 447 | -0.64939 | 0.006158 |
| *Adipoq* | adiponectin | 11 | 49 | -1.98273 | 0.025752 |
| *Slc4a1* | solute carrier family 4  (anion exchanger) | 61 | 179 | -1.49132 | 0.038394 |
| *Ngp* | neutrophilic granule protein | 3 | 24 | -3.285 | 0.042991 |

The table shows the mean numbers of reads for each transcript at day one (i.e. 24 h after being explanted) of culture in control (i.e. fed basal media only) ureters and organs exposed to exogenous TGFβ1 (5 ng/mL). The log2 values of the ratios of the TGFβ1 exposed organs to the controls are shown. The p values have been adjusted for multiple comparisons. n=3 for each experimental set.

**Table S3. Transcripts encoding FGF family members and their receptors, as detected and quantified in the RNA-sequencing analyses**

| **RNA**  **name** | **Encoded molecule** | **Control**  **day one** | **Control**  **day six** | **TGFβ1**  **day one** | **TGFβ1**  **day six** |
| --- | --- | --- | --- | --- | --- |
| **Ligands** | | | | | |
| \| *Fgf1* \|  \| \| --- \| --- \| | fibroblast growth factor 1 | 709 | 2424 | 651 | 2512 |
| *Fgf2* | fibroblast growth factor 2 | 822 | 999 | 872 | 1122 |
| *Fgf3* | fibroblast growth factor 3 | 4 | 3 | 5 | 2 |
| *Fgf4* | fibroblast growth factor 4 | 0 | 0 | 0 | 0 |
| *Fgf 5* | fibroblast growth factor 5 | 19 | 9 | 18 | 12 |
| *Fgf6* | fibroblast growth factor 6 | 0 | 0 | 0 | 0 |
| *Fgf7* | fibroblast growth factor 7 | 494 | 597 | 567 | 791 |
| *Fgf8* | fibroblast growth factor 8 | 0 | 1 | 2 | 1 |
| *Fgf9* | fibroblast growth factor 9 | 34 | 46 | 36 | 46 |
| *Fgf10* | fibroblast growth factor 10 | 1069 | 1419 | 602^a^ | 1136 |
| *Fgf11* | fibroblast growth factor 11 | 322 | 135 | 300 | 176 |
| *Fgf12* | fibroblast growth factor 12 | 42 | 10 | 33 | 9 |
| *Fgf13* | fibroblast growth factor 13 | 173 | 168 | 226 | 175 |
| *Fgf14* | fibroblast growth factor 14 | 138 | 40 | 121 | 64 |
| *Fgf15* | fibroblast growth factor 15 | 0 | 1 | 0 | 2 |
| *Fgf16* | fibroblast growth factor 16 | 9 | 6 | 17 | 14 |
| *Fgf17* | fibroblast growth factor 17 | 3 | 2 | 4 | 5 |
| *Fgf18* | fibroblast growth factor 18 | 27 | 8 | 172^b^ | 143^c^ |
| *Fgf20* | fibroblast growth factor 20 | 27 | 13 | 19 | 13 |
| *Fgf21* | fibroblast growth factor 21 | 2 | 13 | 5 | 17 |
| *Fgf22* | fibroblast growth factor 22 | 0 | 0 | 1 | 1 |
| *Fgf23* | fibroblast growth factor 23 | 0 | 0 | 0 | 0 |

**Table S3 (continued)**

| **Receptors** | | | | | |
| --- | --- | --- | --- | --- | --- |
| *Fgfr1* | fibroblast growth factor receptor 1 | 4372 | 2971 | 5612 | 4527 |
| *Fgfr2* | fibroblast growth factor receptor 3 | 6116 | 3607^d^ | 5583 | 4110 |
| *Fgfr3* | fibroblast growth factor receptor 3 | 2948 | 3159 | 2379 | 2601 |
| *Fgfr4* | fibroblast growth factor receptor 4 | 257 | 159 | 239 | 151 |

The table shows the mean numbers of reads for each transcript at day one and six of culture in control (i.e. fed basal media only) ureters and organs exposed to exogenous TGFβ1 (5 ng/mL). ^a^Control day one versus TGFβ1 day one p=0.0052; ^b^control day one versus TGFβ1 day one p=6.55E-13; ^c^control day six versus TGFβ1 day six p=0.0361; ^d^control day one versus control day six p=0.0013. n=3 for each experimental set.
